# Environmental effects on genetic variance are likely to constrain adaptation in novel environments

**DOI:** 10.1093/evlett/qrad065

**Published:** 2024-01-18

**Authors:** Greg M Walter, Keyne Monro, Delia Terranova, Enrico la Spina, Maria Majorana, Giuseppe Pepe, James Clark, Salvatore Cozzolino, Antonia Cristaudo, Simon J Hiscock, Jon Bridle

**Affiliations:** School of Biological Sciences, University of Bristol, Bristol, United Kingdom; School of Biological Sciences, Monash University, Melbourne, Australia; School of Biological Sciences, Monash University, Melbourne, Australia; Department of Biology, University of Naples Federico II, Naples, Italy; Department of Biological, Geological and Environmental Sciences, University of Catania, Catania, Italy; Department of Biological, Geological and Environmental Sciences, University of Catania, Catania, Italy; Department of Biological, Geological and Environmental Sciences, University of Catania, Catania, Italy; School of Biological Sciences, University of Bristol, Bristol, United Kingdom; Department of Biology, University of Oxford, Oxford, United Kingdom; Department of Biology, University of Naples Federico II, Naples, Italy; Department of Biological, Geological and Environmental Sciences, University of Catania, Catania, Italy; Department of Biology, University of Oxford, Oxford, United Kingdom; School of Biological Sciences, University of Bristol, Bristol, United Kingdom; Department of Genetics, Evolution and Environment, University College London, London, United Kingdom

**Keywords:** additive genetic variance, covariance tensor, G-matrix, novel environments, phenotypic plasticity, *Senecio*

## Abstract

Adaptive plasticity allows populations to cope with environmental variation but is expected to fail as conditions become unfamiliar. In novel conditions, populations may instead rely on rapid adaptation to increase fitness and avoid extinction. Adaptation should be fastest when both plasticity and selection occur in directions of the multivariate phenotype that contain abundant genetic variation. However, tests of this prediction from field experiments are rare. Here, we quantify how additive genetic variance in a multivariate phenotype changes across an elevational gradient, and test whether plasticity and selection align with genetic variation. We do so using two closely related, but ecologically distinct, sister species of Sicilian daisy (*Senecio*, Asteraceae) adapted to high and low elevations on Mt. Etna. Using a quantitative genetic breeding design, we generated and then reciprocally planted c. 19,000 seeds of both species, across an elevational gradient spanning each species’ native elevation, and then quantified mortality and five leaf traits of emergent seedlings. We found that genetic variance in leaf traits changed more across elevations than between species. The high-elevation species at novel lower elevations showed changes in the distribution of genetic variance among the leaf traits, which reduced the amount of genetic variance in the directions of selection and the native phenotype. By contrast, the low-elevation species mainly showed changes in the amount of genetic variance at the novel high elevation, and genetic variance was concentrated in the direction of the native phenotype. For both species, leaf trait plasticity across elevations was in a direction of the multivariate phenotype that contained a moderate amount of genetic variance. Together, these data suggest that where plasticity is adaptive, selection on genetic variance for an initially plastic response could promote adaptation. However, large environmental effects on genetic variance are likely to reduce adaptive potential in novel environments.

## Introduction

Populations facing rapid environmental change must cope with increasingly novel environments if they are to persist. Plastic responses to different environments can aid persistence by allowing populations to rapidly change their mean phenotypes to track changes in phenotypic optima, maintaining fitness in each environment (Charmantier et al., 2008; Via et al., 1995). However, plasticity should only evolve to track environments that populations regularly experience (Ashander et al., 2016; Chevin et al., 2010; Hermisson & Wagner, 2004) and is unlikely to maintain fitness in novel environments because plastic responses have not evolved to suit those environments (Acasuso-Rivero et al., 2019; Palacio-López et al., 2015). When plasticity fails to maintain fitness, genotypes can vary in the extent to which they suffer reduced fitness, increasing the potential for populations to recover fitness when selection on ecologically important traits leads to adaptation (Shaw & Shaw, 2014; Walter et al., 2023). Our understanding of the potential for adaptation in novel environments is limited because field experiments that measure plasticity, selection, and genetic variation along ecological gradients are rare.

**Figure 1.**
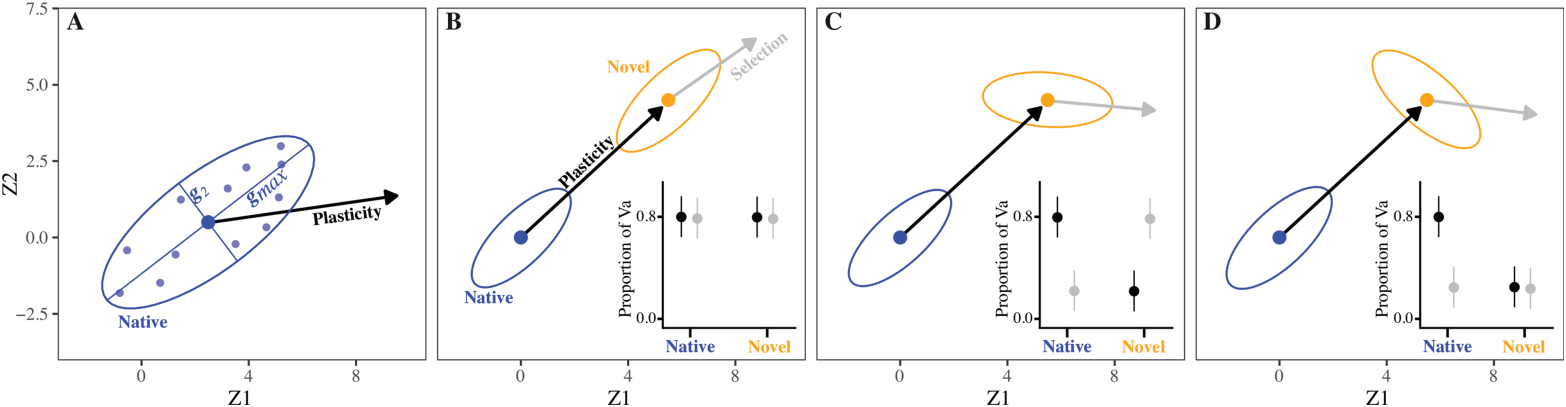
(A) A genetic correlation between two traits (Z1 and Z2) concentrates genetic variation in the traits (summarized by the additive genetic variance–covariance matrix, **G**) along axes defined by trait combinations. The ellipse shows genetic variation in the traits as a cloud of genotypes (small circles) in multivariate space with ***g***_max_ representing the direction in which both traits vary most genetically. In this case, genotypes with larger values of Z1 also have larger values of Z2. Plasticity is the change in trait mean (large circle) in response to a change in environment. (B–D) Comparing plasticity and selection with genetic variation for Z1 and Z2 in a population at its native elevation (blue) and a novel elevation (orange). Again, ellipses show genetic variation in traits and solid circles within ellipses are trait means. Black arrows are changes in trait means across environments due to plasticity, and gray arrows are the directions of selection at the novel elevation. Insets show the proportions of genetic variation in traits in the direction of plasticity and selection in each environment. Adaptation will be fastest if both plasticity and selection are in a direction with a large amount of genetic variance in the traits (i.e., ***g***_max_), as in (B). However, genotype-by-environment interactions (G × E) mean that genotypes vary in plasticity, which can change genetic variation in traits across environments, as in (C–D). In the novel environment, if plasticity creates phenotypes that differ to those favored by selection, then plasticity will be nonadaptive. In this case, for rapid adaptation to be possible, G × E interactions would need to increase genetic variation in the direction of selection, as in (C). However, adaptation in the novel environment will be constrained if G × E reduces the amount of genetic variation in the direction of selection in that environment, as in (D).

For adaptation to occur, selection must operate on genetic variation in multiple traits that combine to form a multivariate phenotype (Blows, 2007). However, pleiotropy and linkage often create genetic correlations among traits, meaning that any changes to one trait will enact changes in other traits that are genetically correlated. The additive genetic variance–covariance matrix (**G**) summarizes how genetic variation is distributed among a given set of traits (Figure 1A; Lande, 1979; Walsh & Blows, 2009). The diagonal of **G** captures genetic variation in each trait, while the off-diagonal of **G** measures the covariation between each pair of traits due to shared genetic variation (Steppan et al., 2002). Stronger covariances between traits concentrate the total variance in **G** into certain trait combinations (Figure 1A). These are often found by decomposing **G** into independent axes, akin to principal components, representing directions in which phenotypes vary genetically (Lande, 1979; Walsh & Blows, 2009). The primary axis of **G**, known as ***g***_max_, is the direction of the phenotype in which genetic variation is most abundant and the direction in which phenotypic evolution is expected to occur (Costa e Silva et al., 2020; McGlothlin et al., 2018; Schluter, 1996; Walter, 2023; Zu et al., 2020).

In novel environments, plasticity will be at least partially adaptive if it moves a population’s mean phenotype closer to its new optimum (Ghalambor et al., 2007; Lande, 2009). Evidence from diverse taxa also suggests that plasticity, adaptive or otherwise, can be biased toward the direction of ***g***_max_ because genotypes are expected to vary in the direction of environmental heterogeneity (Draghi & Whitlock, 2012; Lind et al., 2015; Noble et al., 2019). Together, these observations predict that adaptation in novel environments will be fastest if plasticity, selection, and genetic variation for a given phenotype are all aligned (Figure 1B; Lande, 2009; Levis & Pfennig, 2016). However, if plasticity is nonadaptive, which is common (Acasuso-Rivero et al., 2019), then its alignment with ***g***_max_ could deflect evolution away from the direction of selection and thereby constrain adaptation to the new environment. Testing these predictions requires field experiments that use natural populations to assess if plastic responses to novel environments are (1) adaptive and (2) produce phenotypes with abundant genetic variation.

The availability of genetic variation can depend on whether genotypes vary in plasticity, known as genotype-by-environment interaction (G × E) (Josephs, 2018). The presence of G × E in a set of traits means that additive genetic variation for the traits and/or covariation between traits (summarized in **G**) changes across environments (Sgrò & Hoffmann, 2004; Wood & Brodie III, 2015). This can aid adaptation to novel environments when G × E causes genetic variation to increase in the phenotypes favored by selection (Figure 1C). Conversely, G × E that reduces genetic variation in those phenotypes should constrain adaptation to novel environments (Figure 1D; Chevin, 2013). To our knowledge, however, field experiments have not quantified plasticity, selection, and genetic variation along environmental gradients as ecological margins are exceeded. Consequently, we do not yet know whether changes in genetic variance across environments increase the adaptive potential of populations when faced with novel conditions.

**Figure 2.**
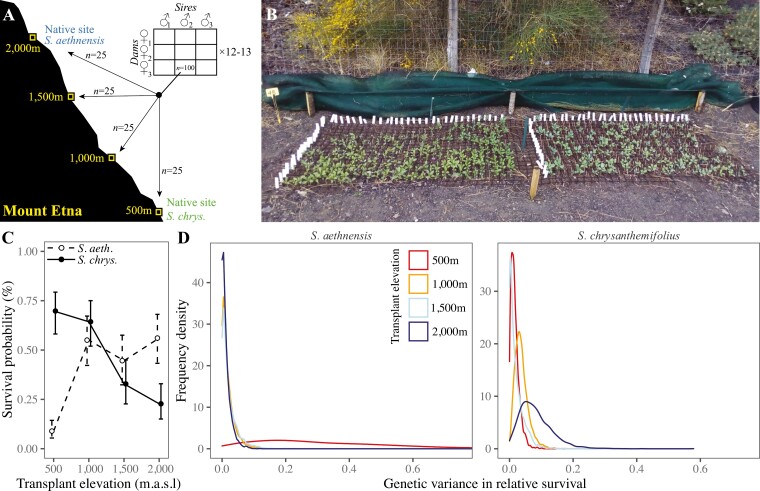
The experimental design. (A) For two Etnean *Senecio* species, we mated three sires to three dams in blocks, with 12–13 blocks in total for each species. We collected 100 seeds from each mating (family) and planted 25 seeds (per family) at each of four elevations representing the native elevation of each species and two intermediate elevations. (B) Photo of an experimental block at 2,000 m, 8 weeks after seeds were sowed (*S. chrysanthemifolius* on left). Panels (C) and (D) show analyses of survival data from this experiment, taken from Walter et al. (2022b). (C) Mean survival (±1 SE) for both species across the elevation gradient as the probability of surviving (after seedling establishment) to the end of summer (24th September). (D) Posterior distributions for estimates of genetic variance in survival, which was greater for both species at their novel elevations.

We planted seeds of two closely related, but ecologically contrasting, sister species of Sicilian daisy (*Senecio*, Asteraceae) across an elevational gradient on Mt. Etna (Figure 2A and B). Both species are obligate outcrossers that share generalist insect pollinators (Walter et al., 2020a). *Senecio aethnensis* is a perennial with entire glaucous leaves and is endemic to lava flows > 2,000 meters above sea level (m.a.s.l.) on Mt. Etna, where individuals grow back each spring after snow cover in winter. By contrast, *Senecio chrysanthemifolius* is a short-lived perennial with dissected leaves and occupies disturbed habitats (e.g., roadsides, vineyards) at 500–1,000 m.a.s.l. on Mt. Etna and across Sicily more broadly. Despite its narrower geographical distribution, *S. aethnensis* has greater genetic diversity than *S. chrysanthemifolius*, suggesting that *S. aethnensis* derives from a larger ancestral population (Chapman et al., 2013). However, *S. chrysanthemifolius* shows greater adaptive plasticity in leaf morphology across elevations than *S. aethnensis* (Walter et al. 2022a).

To estimate additive genetic variance in phenotypes and fitness, we used a breeding design to produce seeds for c. 100 families per species. We then reciprocally planted seeds from each family across an elevational gradient spanning the native ranges of both species and two intermediate elevations. We tracked the survival of emergent seedlings, and measured five ecologically important leaf traits that are known to be plastic (Walter et al., 2022a), correlated with fitness (Walter et al., 2023), and associated with adaptive divergence in other *Senecio* species (Richards et al., 2019; Walter et al., 2020b). Previous analyses of survival data from this experiment show that these two species are adapted to their native environments (Figure 2C) and that increased additive genetic variance in survival improves their adaptive potential when planted in novel environments (Figure 2D; Walter et al., 2022b).

Here, we extend these results by estimating the additive genetic variance–covariance matrix (**G**) for the five leaf traits measured in each species at each elevation, allowing us to compare plasticity, selection, and genetic variation in leaf traits along a natural ecological gradient. We first compare **G** across elevations to test whether G × E causes genetic variation in leaf traits to change along this gradient, predicting that **G** would differ the most between native and more novel elevations. We then quantify how much of the genetic variation in leaf traits lies in the direction of plasticity and selection at novel elevations, predicting that G × E would aid adaptation in novel environments if it increased genetic variation in the direction of phenotypes favored by selection.

## Methods and materials

### Breeding design and field experiment

We briefly describe the field experiment and refer readers to Walter et al. (2022b) for more detail. We collected cuttings from c. 80 individuals per species growing naturally at 2,000–2,600 m.a.s.l for *S. aethnensis* and 526–790 m.a.s.l for *S. chrysanthemifolius.* (Supplementary Table S1 and Supplementary Figure S1). We propagated one cutting per field individual in the glasshouse and randomly assigned the individual as a sire (pollen donor) or dam (pollen receiver). We randomly mated three sires to three dams in full-factorial 3 × 3 blocks, completing 12 blocks for *S. aethnensis* (*n* = 36 sires, *n = *35 dams, *n* = 94 full-sibling families) and 13 blocks for *S. chrysanthemifolius* (*n = *38 sires, *n* = 38 dams, *n* = 108 full-sibling families, with two sires and dams in the last block). We then planted 100 seeds from each family on Mt. Etna at four elevations, including the native elevations of both species (500 and 2,000 m) and two intermediate elevations (1,000 and 1,500 m). Vagrant individuals of both species are occasionally found at the intermediate elevations, suggesting the potential to expand their ranges beyond their current distributions.

On the May 7–8, 2019, we planted 25 seeds per family at each transplant elevation, which we randomized into five experimental blocks (*S. aethnensis n* = 432 seeds/block, *n* = 2,160 seeds/site; *S. chrysanthemifolius n* = 540 seeds/block, *n* = 2,700 seeds/site; total *N* = 19,232 seeds). To prepare each block, we cleared all plant matter and placed a plastic grid (4 cm-square cells) on the ground. We attached each seed to the middle of a toothpick using nondrip super glue, then pushed each toothpick into the soil in each grid cell so the seed sat 1–2 mm below the soil surface. To replicate natural germination conditions, we suspended 90% shade cloth 20 cm above each block and kept seeds moist until germination ceased (2–3 weeks). We then replaced the 90% shade cloth with 40% shade cloth to replicate shade that naturally growing plants are often found under. We recorded seedling emergence, survival, and establishment (whether seedlings produced 10 leaves). The experiment ended in January 2020 when mortality stabilized (Supplementary Figure S2) and plants started growing into each other, increasing competition. This precluded recording further data, including reproductive traits.

### Quantifying leaf traits

When more than 80% of plants had produced 10 leaves at each transplant elevation, we collected the 5th and 6th leaves (from the base of plant) to quantify leaf morphology and pigment content (*N* = 6,454 plants). For leaf morphology, we scanned the leaves (Canoscan 9000F) and used *Lamina* (Bylesjo et al., 2008) to quantify leaf complexity $\left(\frac{{leaf\;perimeter}^{2}}{leaf\;area}\right)$, width of leaf indents (mm), and number of leaf indents standardized by perimeter (indents/mm). We then weighed all leaves per plant and calculated specific leaf area (SLA) as $\frac{leaf\;area}{leaf\;weight}$ (mm^2^/mg). We used a Dualex instrument (Force-A, France) to measure the flavonol content of each leaf in spectral reflectance.

To aid comparison of traits measured on different scales, we mean standardized each trait prior to analysis (Hansen & Houle, 2008). This estimates the mean-standardized genetic variance (evolvability) of each trait, as appropriate for comparison with changes in trait means (plasticity) across elevations. We used R (v.3.6.1; R Core Team, 2021) for all analyses.

### Quantifying plasticity as elevational changes in multivariate phenotype

To quantify species differences in leaf plasticity across elevations, we used a multivariate analysis of variance with the five leaf traits as the multivariate response variable, and with elevation, species, and their interaction as fixed effects. Block within elevation and family within species were error terms for species and elevation, respectively. To visualize differences in plasticity, we estimated the D matrix of differences in mean multivariate phenotype and calculated scores for the first two axes of **D**. Methods for constructing **D** are presented in Supplementary Methods S1.

### Estimating additive genetic variation in leaf traits

To estimate the additive genetic variance–covariance matrix (**G**) for the five leaf traits measured in each species at each elevation, we used the package *MCMCglmm* (Hadfield, 2010) to apply the linear mixed model


$$\begin{array}{l} y_{ijkl}=s_{i}+d_{j\left(i\right)}+b_{k}+e_{l\left(ijk\right)}, \end{array}$$ (1)

where leaf traits are the multivariate response variable $\left(y_{ijkl}\right)$, $s_{i}$ is the *i*th sire, $d_{j\left(i\right)}$ the *j*th dam nested within sire, $b_{k}$ the *k*th block, and $e_{l\left(ijk\right)}$ the residual. We mated blocks of males and females in a full-factorial (North Carolina II) breeding design, which can also partition a sire × dam variance capturing the effects of epistasis and dominance (Lynch & Walsh, 1998). However, analyzing this design using the paternal half-sibling approach above simply pools the sire × dam variance with the residual variance, without inflating the sire variance from which additive genetic variation derives (Schielzeth & Nakagawa, 2013). Both approaches produced equivalent results (Supplementary Methods S2), but we chose the paternal half-sibling approach because it improved model convergence, estimated fewer parameters, and estimated sire variance more precisely.

We applied equation 1 separately to the data for each species at each elevation (*n* = 8). We used chains with burn-ins of 150,000 iterations and thinning intervals of 1,500 iterations, saving 2,000 thinned iterations (Markov Chain Monte Carlo [MCMC] samples) as the posterior distributions for all estimates. We confirmed model convergence by checking that chains mixed sufficiently well, that autocorrelations among samples were < 0.05, and that our parameter expanded prior was uninformative (Hadfield, 2010). For each model, we constructed **G** with the sire variances and covariances, which represent one quarter of the additive genetic variation in leaf traits (Lynch & Walsh, 1998).

Since *MCMCglmm* constrains variance estimates to be positive, we tested the significance of estimates in **G** by comparing them with suitable null distributions created by randomizing offspring among sires and reapplying the model to randomized data. To maintain differences among blocks, we randomized offspring within each block separately. We conducted 1,000 randomizations for each observed **G** and concluded that observed estimates in **G** were significant if posterior means exceeded those from null distributions.

Slower growth at higher elevations meant that many plants died before measurement, which could potentially influence our estimates of genetic variance (Supplementary Figure S2). Nevertheless, we measured multiple offspring from all sires and >90% of full-sibling families (4.5–15.6 offspring per family on average), meaning that estimates are based on the entire pedigree and large sample sizes (*n* = 482–1,683 individuals) (Supplementary Table S2). Families also showed similar levels of mortality before and after measurement (Supplementary Methods S3), reducing the likelihood that estimates of genetic variance (and of plasticity and selection) are biased by differences in early mortality among families.

### Comparing additive genetic variation across elevations and species

To quantify differences in genetic variation in leaf traits across elevations and species, we used eigenanalysis to decompose each **G** into independent axes (eigenvectors) defined by linear combinations of traits, representing directions in which leaf phenotypes vary genetically. As such, eigenvectors describe the orientation of genetic variation in leaf phenotypes expressed by each species at each elevation and have eigenvalues describing the amounts of genetic variation in those phenotypes. We used these descriptors to characterize genetic variation in leaf phenotypes and how genetic variation changes across elevations.

For each G-matrix, we used the total amount of genetic variation in leaf traits to describe its size, the distribution of eigenvalues to describe its shape (more elliptical if variation is more condensed toward certain phenotypes), and eigenvectors to describe its orientation. To compare the orientation of genetic variation between elevations, we calculated the angle between ***g***_max_ estimated at the native elevation and ***g***_max_ estimated at each of the other elevations using


$$\begin{array}{l} \theta^{\circ }=arccos\left(|r|\right)\times \frac{180}{\pi }, \end{array}$$ (2)

where r is the correlation between eigenvectors and *θ*° is the angle between them (Berdal & Dochtermann, 2019; Lind et al., 2015).

We then used a covariance tensor approach to formally compare **G** matrices within a single framework. Briefly, the tensor involves an eigenanalysis of the S-matrix, which contains the variances and covariances of individual elements in **G** across our eight matrices. Decomposing **S** therefore provides (after rearrangement and scaling) a set of independent axes (eigentensors) describing differences in **G** across elevations and species. For more details, see Supplementary Figure S3, Hine et al. (2009), Aguirre et al. (2014) and Walter et al. (2018).

To test the significance of observed eigentensors, we compared them to suitable null distributions created by reapplying equation 1 to data reconstructed from randomized breeding values, simulating changes in **G** due only to random sampling (see supplementary code; Morrissey et al., 2019; Walter, 2023). If observed eigentensors described larger differences in **G** compared to null eigentensors, we concluded that genetic variance for leaf traits differed significantly across elevations and/or species. To identify how each original **G** contributed to such differences, we calculated matrix coordinates. Like principal components scores, coordinates describe correlations between eigentensors and the original matrices, so matrices with larger scores contribute more to overall differences described by an eigentensor.

### Comparing plasticity and selection with genetic variation in leaf traits across elevations

#### Does plasticity occur in directions of phenotype with abundant genetic variation?

For each species, we quantified multivariate plasticity in leaf traits between the native elevation and the other elevations. We first calculated the mean of all five leaf traits at each elevation, then calculated multivariate plasticity across elevations as per Noble et al. (2019) using


$$\begin{array}{l} \Delta {\bar{x}}_{i}={\bar{x}}_{native\;elevation}-{\bar{x}}_{i}, \end{array}$$ (3)

where for each species, $\Delta {\bar{x}}_{i}$ is a vector of differences in trait means (for all five leaf traits) between the native elevation $\left({\bar{x}}_{native\;elevation}\right)$ and the *i*th novel elevation $\left({\bar{x}}_{i}\right)$.

At each elevation, we then quantified how much genetic variation was associated with plasticity in leaf traits using the matrix projection


$$\begin{array}{l} V_{ij}=\frac{\Delta {{\bar{x}}_{i}}^{T}G_{ij}\;\Delta {\bar{x}}_{i}}{\;{\lambda_{g_{max}}}_{ij}}, \end{array}$$ (4)

where for each species, $\Delta {\bar{x}}_{i}$ is the plasticity vector from equation 3, *T* is its transpose, and $G_{ij}$ is the *j*th MCMC sample of **G** at the *i*th novel elevation. The projection is divided by ${\lambda_{g_{max}}}_{ij}$ (the eigenvalue of ***g***_max_) to estimate genetic variance in the direction of leaf plasticity as a proportion of the maximum genetic variation available (Noble et al., 2019). Comparing genetic variance between native and novel elevations tests whether changes in **G** (due to G × E) reduce or increase genetic variation in the direction of plasticity.

#### Does selection favor phenotypes with abundant genetic variation in more novel environments?

To estimate viability selection on leaf traits, we calculated phenotypic selection gradients (***β***) by relating traits to survival (our fitness proxy) at each elevation. We could only estimate selection where high mortality occurred after leaf measurements were taken. This is because we could only measure leaves of seedlings that established, which excludes seedlings that died beforehand, and also because without sufficient mortality after measurement there would not be variation in survival with which to link to the measured phenotypes. High premeasurement mortality occurred at 2,000 m (where both species grew more slowly), and for *S. chrysanthemifolius* at elevations above 500 m (Supplementary Figure S2). We were therefore limited to estimating selection on leaf traits of *S. aethnensis* at elevations below 2,000 m where mortality occurred after measurement, acknowledging that such selection may vary over time, and that fecundity selection could also act on traits. To estimate ***β***, we applied a multiple logistic regression using the package *lme4* (Bates et al., 2015) to calculate


$$\begin{array}{l} \beta =P^{-1}s, \end{array}$$ (5)

where s is survival to the end of summer (1 if seedlings survived or 0 if they did not) and **P** is the phenotypic (co)variance matrix of the five mean-standardized traits (Lande & Arnold, 1983). We extracted ***β*** as the vector of partial regression coefficients, transformed them from the logit link scale to the probability response scale, then divided them by mean survival to represent selection acting via relative fitness (Janzen & Stern, 1998). Scaled to unit length, ***β*** estimates the direction of viability selection on leaf traits for *S. aethnensis* at each elevation.

To test whether selection on leaf traits of *S. aethnensis* favors phenotypes with abundant genetic variation at novel lower elevations, we used equation 4 to project the selection vectors (***β***) estimated at 500 m, 1,000 and 1,500 m through the corresponding G matrices. This projection yields the proportion of genetic variation in leaf traits lying in the direction of selection on traits. To test whether elevational changes in **G** increased genetic variation in the direction of selection, we also projected the selection vectors through **G** estimated at the native 2,000 m elevation of *S. aethnensis*. This tested how much genetic variation lay in the direction of selection at novel elevations, relative to the native elevation (as if **G** remained unchanged across elevations). We incorporated uncertainty in estimates of selection by creating 1,000 bootstrap samples of ***β*** using the *boot* package (Canty & Ripley, 2022), then projecting each bootstrapped sample through each MCMC sample of **G**.

#### How abundant is genetic variation in the directions of native phenotypes at novel elevations?

Last, we tested whether genetic variation in leaf traits at novel elevations was abundant in the directions of native phenotypes adapted to those elevations. We quantified the proportion of genetic variation in traits of *S. aethnensis* in the direction of the native phenotype of *S. chrysanthemifolius* at 500 m, and genetic variation in traits of *S. chrysanthemifolius* in the direction of the native phenotype of *S. aethnensis* at 2,000 m. We estimated the directions of native phenotypes by modifying equation 3 to calculate differences in trait means between species at each elevation. We then used equation 4 to project this vector through **G** for *S. aethnensis* at 500 m, and for *S. chrysanthemifolius* at 2,000 m, quantifying the proportion of genetic variation in leaf traits in the directions of native phenotypes at novel elevations.

## Results

### Species differ in plastic responses of leaf traits to elevation

**Figure 3.**
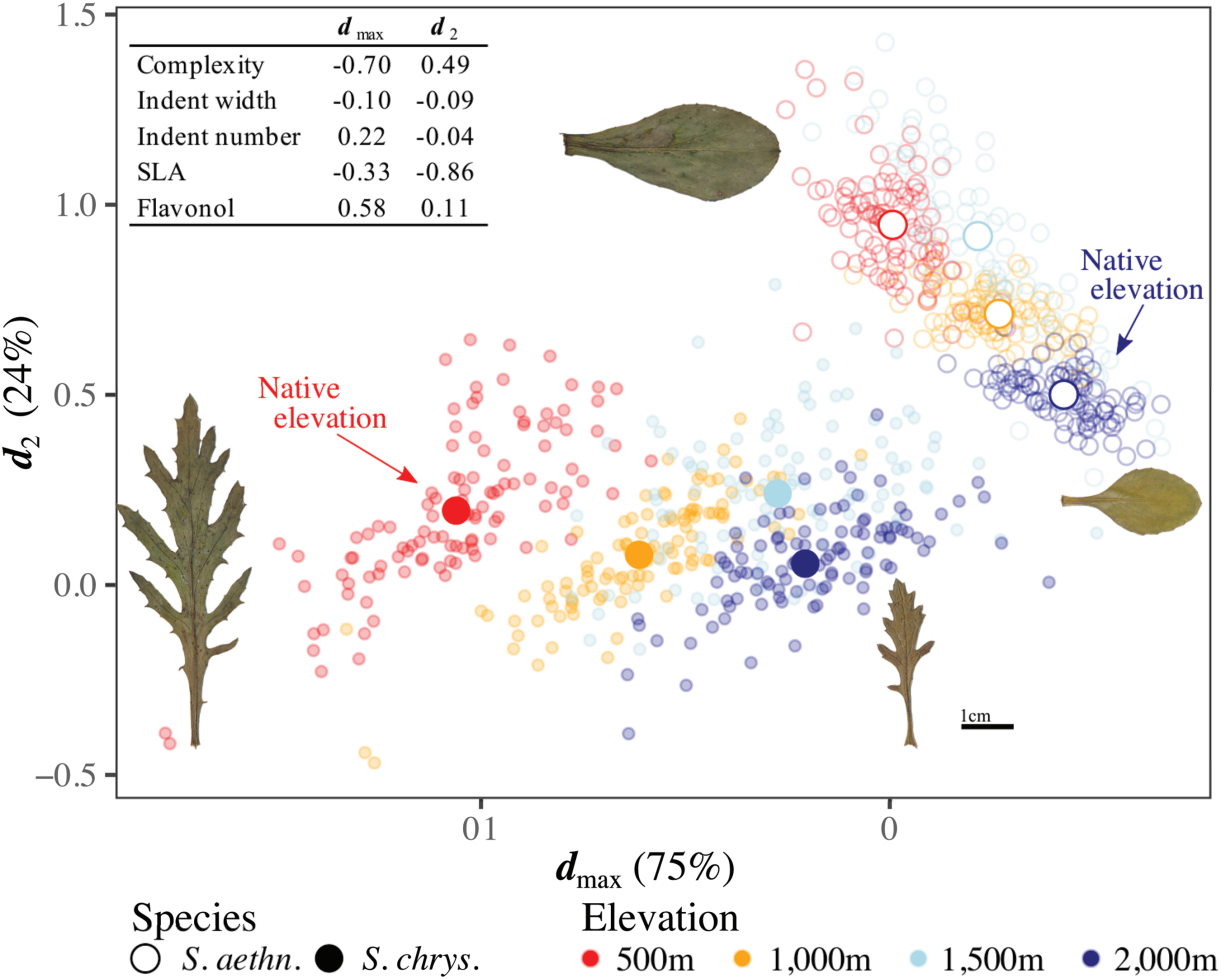
Leaf phenotypes expressed by each species at each elevation. Large, colored circles are species’ multivariate means at each elevation (circles exceed one standard error of the mean), and leaf images show the corresponding mean phenotype at the elevational extremes for both species. Changes in mean phenotype across elevations are due to plasticity, which differs between species. Small circles are full-sibling family means (note that they do not accurately describe genetic variation in phenotypes). Mean phenotypes are calculated using the **D** matrix (see details in text), with the first two axes of **D** (***d***_max_ and ***d***_2_, inset) summarizing 99% of all differences in mean phenotypes across elevations and species. Trait loadings on each axis show how each leaf trait contributes to those differences.

Species differed significantly in the multivariate plasticity of leaf traits across elevations (Figure 3, species × elevation Wilks’ *λ* = 0.79, *F*_3,3011_ = 49.8, *p* < .0001). At 2,000 m, plasticity moved the mean phenotype of *S. chrysanthemifolius* toward the native phenotype of *S. aethnensis*. At 500 m, however, plasticity moved the mean phenotype of *S. aethnensis* away from the native phenotype of *S. chrysanthemifolius* (Figure 3). This suggests that leaf plasticity is adaptive for *S. chrysanthemifolius* at 2,000 m, but nonadaptive for *S. aethnensis* at 500 m. Trait means are presented in Supplementary Figure S4.

### Genetic variation in leaf traits changes more across elevations than between species

**Figure 4.**
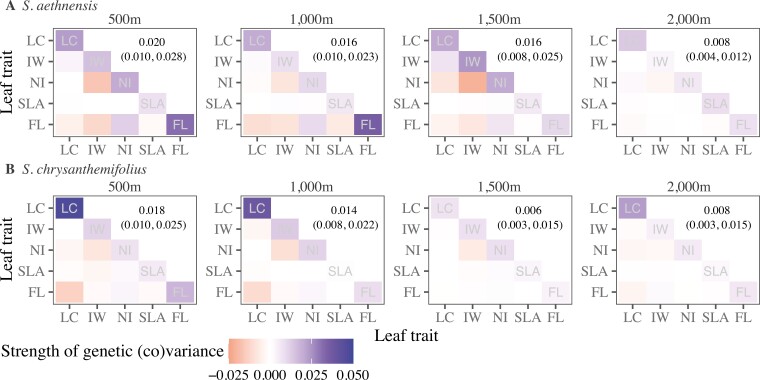
Changes in genetic variation in leaf traits of (A) *S. aethnensis* and (B) *S. chrysanthemifolius* across elevations, visualized as heat maps with elevation increasing left to right. Genetic variances of traits are on the diagonals, with the total genetic variance in each matrix presented above the diagonal (95% highest posterior density intervals in parentheses). Genetic covariances between pairs of traits are below the diagonals. Darker colors indicate stronger variances or covariances, with negative values in red and positive values in blue. Overall, genetic variation in leaf traits differs most between 2,000 m and all lower elevations for *S. aethnensis*, and between 1,500 m and all other elevations for *S. chrysanthemifolius*. LC = leaf complexity; IW = indent width; NI = number of indents; SLA = specific leaf area; FL = flavonol content.

For both species, estimates of additive genetic variation in leaf traits were significant at each elevation (except for SLA in *S. chrysanthemifolius* at high elevations; Supplementary Figure S5) and tended to be lower at 2,000 m than other elevations (Supplementary Figure S6). Changes in **G** across elevations are summarized in Figure 4, which shows that total genetic variation was reduced at higher elevations for both species, and covariances weakened more at higher elevations for *S. aethnensis* than *S. chrysanthemifolius.* G matrices are presented in Supplementary Table S3.

The first two eigenvectors of **G** described 62%–91% of total genetic variation in leaf traits (Table 1), exceeding expectations under the null distribution (Supplementary Figure S7) and demonstrating that axes of **G** are statistically significant. At most elevations, ***g***_max_ (the primary axis of **G**) described >50% of total genetic variation in traits of each species, suggesting strong genetic correlations among traits (Table 1). At 2,000 m, however, ***g***_max_ described 58% genetic variation in traits of *S. chrysanthemifolius* compared to 37% for *S. aethnensis*, suggesting weaker genetic correlations among traits of *S. aethnensis* at its native elevation.

**Table 1.** The first two eigenvectors of **G** (***g***_**max**_ and ***g***_**2**_) for *S. aethnensis* (high-elevation species) and *S. chrysanthemifolius* (low-elevation species) at each transplant elevation.

	500 m		1,000 m		1,500 m		2,000 m	
	*g*_max_	*g*_2_	*g*_max_	*g*_2_	*g*_max_	*g*_2_	*g*_max_	*g*_2_
*S. aethnensis*								
**Eigenvalue**	0.051	0.023	0.047	0.017	0.051	0.016	0.015	0.010
**HPD**	0.029, 0.090	0.013, 0.038	0.026, 0.082	0.009, 0.029	0.025, 0.092	0.007, 0.031	0.009, 0.032	0.005, 0.018
**Proportion (%)**	52	23	59	22	63	20	37	25
**Trait loading**								
Complexity	−0.19	**−0.93**	**−0.35**	**−0.89**	**−0.37**	**−0.92**	**−0.92**	0.11
Indent width	**−0.48**	0.14	**−0.27**	**0.27**	**−0.65**	**0.33**	0.10	−0.04
Indent no.	**0.52**	**−0.33**	**0.29**	**−0.30**	**0.61**	**−0.21**	**−0.28**	−0.05
SLA	−0.03	0.03	−0.15	0.17	0.02	−0.06	0.07	**−0.80**
Flavonol	**0.67**	0.09	**0.84**	−0.14	**0.27**	0.04	**0.25**	**0.58**
*S. chrysanthemifolius*								
**Eigenvalue**	0.053	0.019	0.043	0.023	0.016	0.008	0.024	0.009
**HPD**	0.026, 0.091	0.011, 0.032	0.021, 0.078	0.01, 0.034	0.005, 0.039	0.001, 0.015	0.008, 0.058	0.001, 0.018
**Proportion (%)**	59	22	59	32	50	25	58	22
**Trait loading**								
Complexity	**0.92**	0.18	**0.95**	0.00	−0.08	**0.97**	**0.96**	0.07
Indent width	0.04	**−0.73**	−0.10	**−0.71**	**0.69**	−0.06	−0.08	**0.57**
Indent no.	−0.10	**0.57**	0.01	**0.66**	**−0.69**	−0.16	−0.15	**−0.64**
SLA	−0.02	**0.24**	−0.03	0.00	−0.17	0.05	0.04	−0.18
Flavonol	**−0.36**	**0.21**	**−0.30**	**0.24**	−0.11	−0.18	**−0.23**	**0.47**

*Note*. Eigenvectors represent directions in which leaf traits vary genetically. Trait loadings >0.2 (in bold) are interpreted as contributing substantially to each eigenvector. Eigenvalues and their 95% highest posterior density (HPD) intervals estimate additive genetic variation in these directions, which summarize 62%–91% of all variation in **G** in each case.

For *S. aethnensis*, the combination of leaf traits represented by ***g***_max_ differed between its native elevation (2,000 m) and lower elevations (Table 1). Large angles between ***g***_max_ at native and lower elevations (2,000–1,500 m = 77.68° [39.95, 89.97 highest posterior density (HPD)]; 2,000–1,000 m = 80.91° [34.81, 89.93 HPD]; 2,000–500 m = 83.91° [36.99, 89.96 HPD]) also supported large changes in the orientation of genetic variation in traits across elevations. For *S. chrysanthemifolius*, by contrast, ***g***_max_ represented a similar trait combination at all elevations except 1,500 m (Table 1). This corresponded to small angles between ***g***_max_ at the native elevation (500 m) and both 1,000 m (11.89° [4.16, 56.16 HPD]) and 2,000 m (22.30° [7.94, 66.33 HPD]), but a larger angle between ***g***_max_ at the native elevation and 1,500 m (84.24° [35.49, 90.00 HPD]).

**Figure 5.**
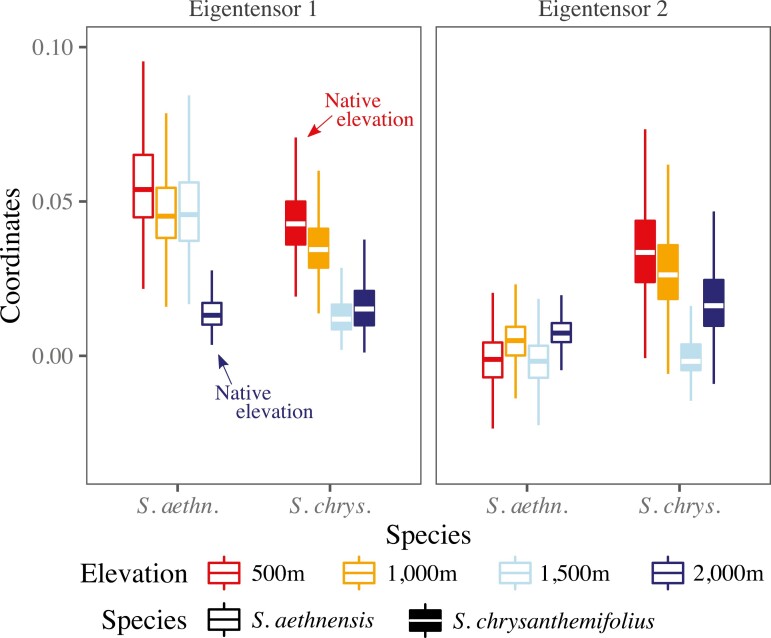
Genetic variation in leaf traits differs more across elevations than between species. Eigentensors summarize differences in **G** across elevations and between species, while coordinates indicate how much each original matrix contributes to the differences described by each eigentensor. Boxplots are posterior distributions for the matrix coordinates. Eigentensor 1 describes large elevational differences, but relatively small species differences, in genetic variation in leaf traits. Eigentensor 2 describes large species differences in genetic variation in leaf traits, which is higher at the lower elevations.

Three (of seven) eigentensors captured significant differences in **G** across species and elevations (Supplementary Figure S8 and Supplementary Table S4). The first eigentensor (E1, 37.6% of all differences in **G**) described large differences in genetic variation in leaf traits across elevations, but only small differences between species (Figure 5). By contrast, the second eigentensor (E2, 28.1% of all differences in **G**) described large differences in genetic variation between species, but smaller differences across elevations (Figure 5). Hence, genetic variation in traits differed more in response to elevation than to adaptive divergence between species. The third eigentensor (E3, 16% of all differences in **G**) described small differences between the two intermediate elevations (not shown).

### Genetic variation in leaf traits is moderate in the direction of plasticity

**Figure 6.**
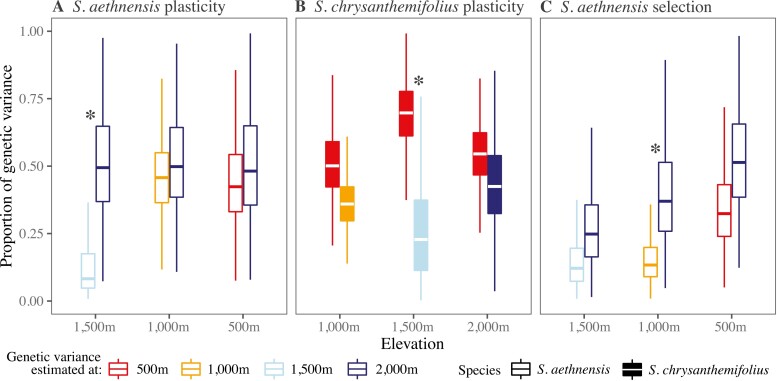
Boxplots of posterior distributions for the proportions of genetic variation in leaf traits that lie in the directions of elevational plasticity (A and B) in those traits and viability selection (C) on those traits in *S. aethnensis*. Note that transplant elevations are ordered by proximity to the native elevation. If plasticity and selection are in directions with higher genetic variation at the native elevation than at other elevations, then G × E reduces genetic variation in those directions at other elevations. For both *S. aethnensis* (A) and *S. chrysanthemifolius* (B), plasticity changed the phenotype in a direction containing more genetic variation at the native elevation compared to more novel elevations. (C) For *S. aethnensis*, only a small proportion (<25%) of genetic variance lay in the direction of selection. Asterisks denote significant differences between genetic variations at the native elevation vs. other elevations (distributions do not overlap at >90% HPD).

For *S. aethnensis*, c. 50% of the maximum genetic variation available in leaf traits at each elevation lay in the direction of leaf plasticity across elevations, except at 1,500 m, where only c. 10% of genetic variation lay in the direction of plasticity (Figure 6A). For *S. chrysanthemifolius*, c. 50%–70% of the maximum genetic variation available lay in the direction of plasticity, and plasticity was consistently associated with more genetic variation at the native elevation than other elevations (Figure 6B). For both species, therefore, leaf traits were plastic in directions containing moderate amounts of genetic variation.

### Changes in elevation reduces genetic variation in the direction of viability selection

Viability selection on leaf traits in *S. aethnensis* favored greater SLA (*χ*^*2*^ = 1.28, *p* < .01) and flavonol content (*χ*^*2*^ = 2.00, *p* < .01) at 1,500 m, greater leaf complexity (*χ*^*2*^ = 1.71, *p* < .01) and SLA (*χ*^*2*^ = 3.00, *p* < .01) at 1,000 m, and greater leaf complexity (*χ*^*2*^ = 132.46, *p* < .01) and flavonol content (*χ*^*2*^ = 41.50, *p* < .01) at 500 m (Supplementary Table S5 and Supplementary Figure S9). Tests had one degree of freedom.

At lower elevations (500–1,500 m), only a small proportion (<25%) of genetic variation in leaf traits of *S. aethnensis* lay in the direction of viability selection on the same traits (***β***) (Figure 6C). Moreover, more genetic variation lay in the direction of selection at the native elevation than other elevations, suggesting that genetic variation in the direction of viability selection changes across elevations (Figure 6C). Genotypic selection gradients, estimated using family means for traits and survival, gave similar results (Supplementary Figure S10).

### Genetic variation in the direction of the native leaf phenotype is abundant for only one species

**Figure 7.**
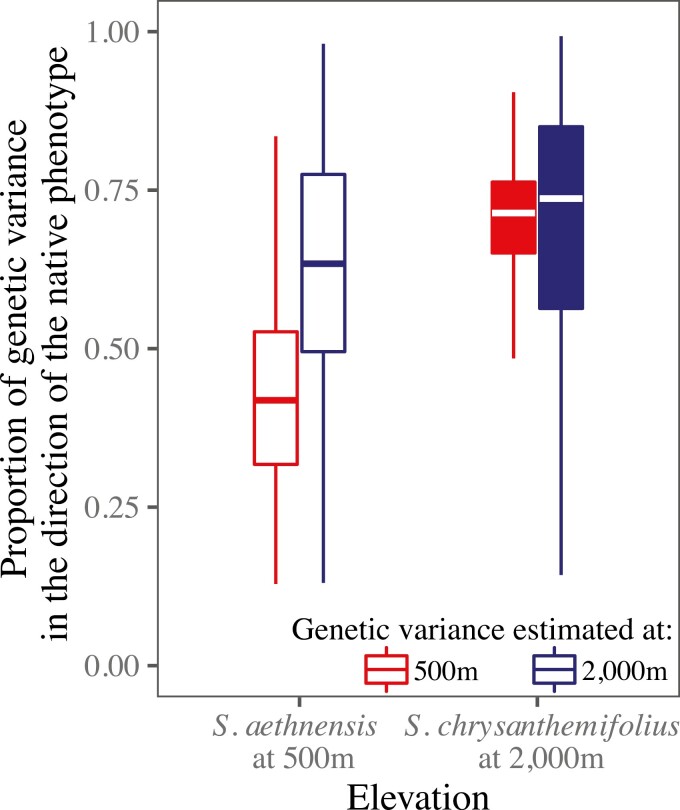
Boxplots of posterior distributions for the proportions of genetic variation in leaf traits that lie in the direction of the native phenotype of each species planted at both native elevations. At 500 m, genetic variation in leaf traits of *S. aethnensis* aligns more poorly with the native phenotype of *S. chrysanthemifolius*, when compared to genetic variance estimated at the native 2,000 m elevation. At 2,000 m, genetic variation in leaf traits of *S. chrysanthemifolius* (estimated at both the native and novel elevations) align more closely with the native phenotype of *S. aethnensis*.

When *S. aethnensis* was planted at the novel 500 m elevation, c. 40% of genetic variation in leaf traits lay in the direction of the native phenotype of *S. chrysanthemifolius*, which was c. 20% less than variation in this direction when *S. aethnensis* was at its native elevation of 2,000 m (Figure 7). Yet when *S. chrysanthemifolius* was planted at a novel elevation of 2,000 m, c. 70% of genetic variation lay in the direction of the native phenotype of *S. aethnensis*, and differed little from when *S. chrysanthemifolius* was at its native elevation of 500 m (Figure 7). Hence, for *S. aethnensis*, G × E from native to novel elevations reduced genetic variation in the direction of the native phenotype adapted to 500 m. For *S. chrysanthemifolius*, however, G × E was weaker and more genetic variation lay in the direction of the native phenotype adapted to 2,000 m.

## Discussion

Field studies combining estimates of plasticity, selection, and genetic variation are exceedingly rare. We generated seeds for two closely related but ecologically distinct Sicilian *Senecio* species, which we reciprocally planted across an elevational gradient, and then estimated plasticity, viability selection, and genetic variation for five ecologically important leaf traits on emergent seedlings. Species differed in leaf plasticity (Figure 3), which showed more evidence of being adaptive in *S. chrysanthemifolius* (Walter et al. 2022a). Higher elevations were associated with less genetic variation in leaf phenotypes (Figure 4) and meant that genetic variation in the leaf traits differed more across elevations than between species (Figure 5). Genetic correlations among traits changed less across elevations for *S. chrysanthemifolius* than *S. aethnensis*, which meant that genetic variation in leaf phenotypes changed less across elevations for *S. chrysanthemifolius* than *S. aethnensis.* For both species, plasticity across elevations produced leaf phenotypes that contained moderate amounts (50%–60%) of the genetic variation available (Figure 6). For *S. aethnensis*, novel lower elevations reduced genetic variation in the direction of selection on the leaf traits (Figure 6C) and the native phenotype of *S. chrysanthemifolius* at 500 m (Figure 7). By contrast, *S. chrysanthemifolius* showed greater genetic variation in the direction of the native phenotype of *S. aethnensis* at 2,000 m (Figure 7). These results suggest that large G × E across environments is likely to reduce genetic variation in the direction of selection in novel environments, as shown by *S. aethnensis* exposed to novel low elevations (reflecting the scenario in Figure 1D). By contrast, smaller G × E could be more beneficial in novel environments by maintaining (to some extent) genetic variation in the direction of selection, as shown by *S. chrysanthemifolius* exposed to novel high elevations (reflecting the scenario in Figure 1B). Our results therefore suggest that nonadaptive plasticity and large environmental effects on genetic variance introduce genetic constraints on adaptation in novel environments, but the extent to which G × E is nonadaptive is likely to be species specific.

### Moderate amounts of genetic variance in the direction of plasticity

We found moderate amounts of genetic variance in the direction of plasticity, providing some support for a meta-analysis that suggested plasticity is biased in directions of phenotypes with large amounts of additive genetic variance (Noble et al., 2019). In this meta-analysis, however, the amount of genetic variance in the direction of plasticity varied across species, traits, and environments. The alignment between plasticity and genetic variation could differ among studies because species (and populations) vary in their amount of genetic variation in plasticity. During adaptation, if the environment is predictable (as required for adaptive plasticity to evolve; Leung et al., 2020) and selection on plasticity is strong, then adaptation is likely to erode genetic variation in plasticity (Hoffmann & Bridle, 2022; Oostra et al., 2018), which could bias plasticity in the direction of historical selection rather than that of contemporary genetic variation. This could explain our observation of only moderate amounts of genetic variance in the direction of plasticity. Resolving how and when genetic variation aligns with plasticity (and understanding whether plasticity is adaptive) will reveal when selection on existing plastic responses could promote adaptation to novel environments. Regardless, our results suggest that if genetic variation aligns with plasticity that is nonadaptive (as was the case for *S. aethnensis*), then plasticity is likely to create genetic constraints that prevent rapid adaptation to novel environments.

### Environmental effects on genetic variance

To better understand the potential for rapid adaptation, it is important to understand how genotypes across a species’ range vary in their responses to environmental variation, and whether populations harbor genetic variation that could help align **G** with selection in novel environments (Bridle & Hoffmann, 2022; Chevin & Lande, 2011; Henry & Stinchcombe, 2023; Lind et al., 2015). Here, G × E produced larger changes in genetic variation in leaf traits across elevations than between two closely related species of *Senecio*. For the high-elevation species, G × E created larger changes in genetic variation in leaf traits that reduced the amount of genetic variation in the direction of selection and the native phenotype at novel low elevations. By contrast, the low-elevation species showed smaller changes in genetic variation at higher elevations that resulted in greater genetic variation in the direction of the native phenotype. Wood and Brodie III (2015) demonstrated that **G** is likely to be affected by the environment as much as by evolution, although it was unclear why. We help to resolve this by demonstrating that novel environments not only change genetic variance in leaf phenotypes more than evolutionary history, but also that such changes can result from nonadaptive genotype-by-environment interactions (G × E) in response to stress when the high-elevation species experience low elevations. Changes in **G** may often be maladaptive in novel environments because plasticity has not evolved to suit those conditions. *S. aethnensis* shows greater genomic diversity than *S. chrysanthemifolius* (Chapman et al. 2013), suggesting that G × E underlying the large elevational changes in genetic variation in leaf traits observed here could be because *S. aethnensis* originated from a larger ancestral population. Given that evolutionary history likely determines G × E, the extent to which G × E is adaptive in novel environments is likely species specific.

### Estimating plasticity and genetic variance when there is mortality before trait measurement

Although traits of both species showed substantial plasticity and changes in genetic variance across elevations, we could not measure traits before high mortality of seedlings, especially at high elevations (1,500–2,000 m). If mortality is nonrandom with respect to phenotype, then selection in early life is likely to target the traits that are either expressed first (potentially cotyledons and first true leaves) or genetically correlated with our measured traits (Weis, 2018). It is therefore possible that mortality before measurement could change the distributions of leaf phenotypes at high elevations, which could have two consequences for our results. First, estimates of leaf plasticity across elevation could be biased if the phenotypes measured at 2,000 m do not reflect the true mean phenotype at that elevation. Note, however, that we found the same patterns of plasticity in a previous experiment on Mt. Etna that bypassed mortality at early life history by transplanting cuttings of both species (Walter et al., 2022a), supporting the results here. Second, estimates of genetic variance could be biased if seedling mortality removed certain phenotypes before traits were measured (Hadfield, 2008). This issue is notoriously difficult to avoid in field experiments where mortality occurs early in life history. However, our estimates should be robust given that individuals from almost all families of *S. chrysanthemifolius*, and all families of *S. aethnensis*, were measured before substantial mortality occurred. Furthermore, families showed similar levels of mortality before and after trait measurement (Supplementary Methods S3) and comparing genetic variance with and without mortality had little effect on the amount and distribution of genetic variance (Supplementary Methods S4). Both of these observations suggest that mortality before measurement should not bias our results.

To adequately replicate families at each elevation, it was necessary to plant seeds close to each other. In natural populations, young seedlings (two to three true leaves) were not observed at high densities and mature plants typically occur >30 cm apart, suggesting that intraspecific competition is generally low. To ensure that our experiment measured traits under conditions that were as ecologically relevant as possible, we focused on early (seedling) leaf traits and avoided traits expressed later when competition increased as plants grew into one another. Our estimates are therefore limited to episodes of selection operating through early survival and exclude later episodes operating during reproduction. Nevertheless, populations experiencing novel environments are likely to incur stronger selection through viability than fecundity, at least during initial colonization (Hadfield, 2008; Mittell & Morrissey, 2023).

## Conclusion

We show that environmental effects on genetic variance in ecologically relevant traits are likely to introduce genetic constraints for adapting to novel environments. When species encounter such environments, the extent to which their existing plasticity in traits is adaptive, and their potential to adapt to the new conditions, will be species specific (Walter et al., 2022a). For both *Senecio* species studied here, plasticity produced leaf phenotypes with moderately high amounts of genetic variation, suggesting that the direction of plasticity will to some extent determine the direction of evolution, and will likely determine the strength of genetic constraints that slow adaptation and make local extinction more likely than evolutionary rescue via rapid adaptation. Genetic variance in leaf traits changed more across elevations for the high-elevation species than the low-elevation species, reducing genetic variation in the direction of selection on leaf traits of the high-elevation species as a result. Therefore, although novel elevations increase adaptive potential by increasing genetic variation in survival (Walter et al., 2022b), the high-elevation species likely faces genetic constraints for adaptation to occur in key leaf traits at lower elevations, and by extension, warmer conditions created by climate change. By contrast, the low-elevation species should adapt faster to higher elevations because its genetic variation in leaf traits aligned better with plasticity and the native phenotype at high elevations. Such variation in responses to warmer vs. cooler environments, even between closely related species, makes predicting adaptation to novel environments challenging. Nevertheless, our results support evidence that limits to plasticity and adaptation are stronger at warmer range margins than cooler margins (Anderson & Wadgymar, 2020; Arnold et al., 2022; Kellermann et al., 2012; Sheth & Angert, 2018; van Heerwaarden et al., 2016). Future studies that manipulate climate without altering other variables could directly test the effect of warming on these two species. In particular, testing the resilience of *S. chrysanthemifolius* to warming would provide insight into the response of lower elevation ecosystems to climate change.

## Supplementary Material

qrad065_suppl_Supplementary_Material

## Data Availability

All data and code can be accessed at Dryad: https://doi.org/10.5061/dryad.k6djh9wdf.

## References

[CIT0001] Acasuso-Rivero, C., Murren, C. J., Schlichting, C. D., & Steiner, U. K. (2019). Adaptive phenotypic plasticity for life-history and less fitness-related traits. Proceedings of the Royal Society B: Biological Sciences, 286(1904), 20190653. 10.1098/rspb.2019.0653 PMC6571476 31185861

[CIT0002] Aguirre, J. D., Hine, E., McGuigan, K., & Blows, M. W. (2014). Comparing G: Multivariate analysis of genetic variation in multiple populations. Heredity, 112(1), 21–29. 10.1038/hdy.2013.12 23486079 PMC3860158

[CIT0003] Anderson, J. T., & Wadgymar, S. M. (2020). Climate change disrupts local adaptation and favours upslope migration. Ecology Letters, 23(1), 181–192. 10.1111/ele.13427 31729141

[CIT0004] Arnold, P. A., Wang, S., Catling, A. A., Kruuk, L. E., & Nicotra, A. B. (2022). Patterns of phenotypic plasticity along a thermal gradient differ by trait type in an alpine plant. Functional Ecology, 36, 2412–2428.

[CIT0005] Ashander, J., Chevin, L. M., & Baskett, M. L. (2016). Predicting evolutionary rescue via evolving plasticity in stochastic environments. Proceedings of the Royal Society B: Biological Sciences, 283(1839), 20161690. 10.1098/rspb.2016.1690 PMC5046909 27655762

[CIT0006] Bates, D., Machler, M., Bolker, B. M., & Walker, S. C. (2015). Fitting linear mixed-effects models using lme4. Journal of Statistical Software, 67, 1–48.

[CIT0007] Berdal, M. A., & Dochtermann, N. A. (2019). Adaptive alignment of plasticity with genetic variation and selection. Journal of Heredity, 110(4), 514–521. 10.1093/jhered/esz022 31259372

[CIT0008] Blows, M. W. (2007). A tale of two matrices: Multivariate approaches in evolutionary biology. Journal of Evolutionary Biology, 20(1), 1–8. 10.1111/j.1420-9101.2006.01164.x 17209986

[CIT0009] Bridle, J., & Hoffmann, A. (2022). Understanding the biology of species’ ranges: When and how does evolution change the rules of ecological engagement? Philosophical Transactions of the Royal Society of London, Series B: Biological Sciences, 377(1848), 20210027. 10.1098/rstb.2021.0027 35184590 PMC8859517

[CIT0010] Bylesjo, M., Segura, V., Soolanayakanahally, R. Y., Rae, A. M., Trygg, J., Gustafsson, P., Jansson, S., & Street, N. R. (2008). LAMINA: A tool for rapid quantification of leaf size and shape parameters. BMC Plant Biology, 8, 82. 10.1186/1471-2229-8-82 18647399 PMC2500018

[CIT0011] Canty, A., & Ripley, B. (2022). boot: Bootstrap R (S-Plus) Functions. R package. https://cran.r-project.org/web/packages/boot/index.html

[CIT0012] Chapman, M. A., Hiscock, S. J., & Filatov, D. A. (2013). Genomic divergence during speciation driven by adaptation to altitude. Molecular Biology and Evolution, 30(12), 2553–2567. 10.1093/molbev/mst168 24077768 PMC3840311

[CIT0013] Charmantier, A., McCleery, R. H., Cole, L. R., Perrins, C., Kruuk, L. E. B., & Sheldon, B. C. (2008). Adaptive phenotypic plasticity in response to climate change in a wild bird population. Science, 320(5877), 800–803. 10.1126/science.1157174 18467590

[CIT0014] Chevin, L. M. (2013). Genetic constraints on adaptation to a changing environment. Evolution, 67(3), 708–721. 10.1111/j.1558-5646.2012.01809.x 23461322

[CIT0015] Chevin, L. M., & Lande, R. (2011). Adaptation to marginal habitats by evolution of increased phenotypic plasticity. Journal of Evolutionary Biology, 24(7), 1462–1476. 10.1111/j.1420-9101.2011.02279.x 21545421

[CIT0016] Chevin, L. M., Lande, R., & Mace, G. M. (2010). Adaptation, plasticity, and extinction in a changing environment: Towards a predictive theory. PLoS Biology, 8(4), e1000357. 10.1371/journal.pbio.1000357 20463950 PMC2864732

[CIT0017] Costa e Silva, J., Potts, B. M., & Harrison, P. A. (2020). Population divergence along a genetic line of least resistance in the tree species *Eucalyptus globulus*. Genes, 11(9), 1095. 10.3390/genes11091095 32962131 PMC7565133

[CIT0018] Draghi, J. A., & Whitlock, M. C. (2012). Phenotypic plasticity facilitates mutational variance, genetic variance, and evolvability along the major axis of environmental variation. Evolution, 66(9), 2891–2902. 10.1111/j.1558-5646.2012.01649.x 22946810

[CIT0019] Ghalambor, C. K., McKay, J. K., Carroll, S. P., & Reznick, D. N. (2007). Adaptive versus non-adaptive phenotypic plasticity and the potential for contemporary adaptation in new environments. Functional Ecology, 21(3), 394–407. 10.1111/j.1365-2435.2007.01283.x

[CIT0020] Hadfield, J. D. (2008). Estimating evolutionary parameters when viability selection is operating. Proceedings of the Royal Society B: Biological Sciences, 275(1635), 723–734. 10.1098/rspb.2007.1013 PMC2596846 18211873

[CIT0021] Hadfield, J. D. (2010). MCMC methods for multi-response generalized linear mixed models: The MCMCglmm R package. Journal of Statistical Software, 33(2), 1–22.20808728

[CIT0022] Hansen, T. F., & Houle, D. (2008). Measuring and comparing evolvability and constraint in multivariate characters. Journal of Evolutionary Biology, 21(5), 1201–1219. 10.1111/j.1420-9101.2008.01573.x 18662244

[CIT0023] Henry, G. A., & Stinchcombe, J. R. (2023). Strong selection is poorly aligned with genetic variation in *Ipomoea hederacea*: Implications for divergence and constraint. Evolution, 77(7), 1712–1719. 10.1093/evolut/qpad078 37105946

[CIT0024] Hermisson, J., & Wagner, G. P. (2004). The population genetic theory of hidden variation and genetic robustness. Genetics, 168(4), 2271–2284. 10.1534/genetics.104.029173 15611191 PMC1448756

[CIT0025] Hine, E., Chenoweth, S. F., Rundle, H. D., & Blows, M. W. (2009). Characterizing the evolution of genetic variance using genetic covariance tensors. Philosophical Transactions of the Royal Society of London, Series B: Biological Sciences, 364(1523), 1567–1578. 10.1098/rstb.2008.0313 19414471 PMC2691006

[CIT0026] Hoffmann, A. A., & Bridle, J. R. (2022). The dangers of irreversibility in an age of increased uncertainty: Revisiting plasticity in invertebrates. Oikos, 00, 1–15.

[CIT0027] Janzen, F. J., & Stern, H. S. (1998). Logistic regression for empirical studies of multivariate selection. Evolution, 52(6), 1564–1571. 10.1111/j.1558-5646.1998.tb02237.x 28565316

[CIT0028] Josephs, E. B. (2018). Determining the evolutionary forces shaping G x E. New Phytologist, 219(1), 31–36. 10.1111/nph.15103 29574919

[CIT0029] Kellermann, V., Overgaard, J., Hoffmann, A., Flogaard, C., Svenning, J. -C., & Loeschcke, V. (2012). Upper thermal limits of *Drosophila* are linked to species distributions and strongly constrained phylogenetically. Proceedings of the National Academy of Sciences of the United States of America, 109, 16228–16233. 22988106 10.1073/pnas.1207553109 PMC3479592

[CIT0030] Lande, R. (1979). Quantitative genetic analysis of multivariate evolution, applied to brain: Body size allometry. Evolution, 33(1Part2), 402–416. 10.1111/j.1558-5646.1979.tb04694.x 28568194

[CIT0031] Lande, R. (2009). Adaptation to an extraordinary environment by evolution of phenotypic plasticity and genetic assimilation. Journal of Evolutionary Biology, 22(7), 1435–1446. 10.1111/j.1420-9101.2009.01754.x 19467134

[CIT0032] Lande, R., & Arnold, S. J. (1983). The measurement of selection on correlated characters. Evolution, 37(6), 1210–1226. 10.1111/j.1558-5646.1983.tb00236.x 28556011

[CIT0033] Leung, C., Rescan, M., Grulois, D., & Chevin, L. M. (2020). Reduced phenotypic plasticity evolves in less predictable environments. Ecology Letters, 23(11), 1664–1672. 10.1111/ele.13598 32869431 PMC7754491

[CIT0034] Levis, N. A., & Pfennig, D. W. (2016). Evaluating “plasticity-first” evolution in nature: Key criteria and empirical approaches. Trends in Ecology and Evolution, 31(7), 563–574. 10.1016/j.tree.2016.03.012 27067134

[CIT0035] Lind, M. I., Yarlett, K., Reger, J., Carter, M. J., & Beckerman, A. P. (2015). The alignment between phenotypic plasticity, the major axis of genetic variation and the response to selection. Proceedings of the Royal Society B: Biological Sciences, 282(1816), 20151651. 10.1098/rspb.2015.1651 PMC4614775 26423845

[CIT0036] Lynch, M., & Walsh, B. (1998). Genetics and analysis of quantitative traits. Sinauer Associates, Inc.

[CIT0037] McGlothlin, J. W., Kobiela, M. E., Wright, H. V., Mahler, D. L., Kolbe, J. J., Losos, J. B., & Brodie, E. D. III. (2018). Adaptive radiation along a deeply conserved genetic line of least resistance in *Anolis* lizards. Evolution Letters, 2(4), 310–322. 10.1002/evl3.72 30283684 PMC6121822

[CIT0038] Mittell, E. A., & Morrissey, M. (2023). The missing fraction problem as an episodes of selection problem. bioRxiv 538558. 10.1101/2023.04.27.538558, preprint: not peer reviewed. 38374726

[CIT0039] Morrissey, M. B., Hangartner, S., & Monro, K. (2019). A note on simulating null distributions for G matrix comparisons. Evolution, 73(12), 2512–2517. 10.1111/evo.13842 31502676

[CIT0040] Noble, D. W. A., Radersma, R., & Uller, T. (2019). Plastic responses to novel environments are biased towards phenotype dimensions with high additive genetic variation. Proceedings of the National Academy of Sciences of the United States of America, 116(27), 13452–13461. 10.1073/pnas.1821066116 31217289 PMC6613099

[CIT0041] Oostra, V., Saastamoinen, M., Zwaan, B. J., & Wheat, C. W. (2018). Strong phenotypic plasticity limits potential for evolutionary responses to climate change. Nature Communications, 9(1), 1–11. 10.1038/s41467-018-03384-9 PMC5843647 29520061

[CIT0042] Palacio-López, K., Beckage, B., Scheiner, S., & Molofsky, J. (2015). The ubiquity of phenotypic plasticity in plants: A synthesis. Ecology and Evolution, 5(16), 3389–3400. 10.1002/ece3.1603 26380672 PMC4569034

[CIT0043] R Core Team. (2021). R: A language and environment for statistical computing. R Foundation for Statistical Computing.

[CIT0044] Richards, T. J., Ortiz-Barrientos, D., & McGuigan, K. (2019). Natural selection drives leaf divergence in experimental populations of *Senecio lautus* under natural conditions. Ecology and Evolution, 9(12), 6959–6967. 10.1002/ece3.5263 31380026 PMC6662321

[CIT0045] Schielzeth, H., & Nakagawa, S. (2013). Nested by design: Model fitting and interpretation in a mixed model era. Methods in Ecology and Evolution, 4(1), 14–24. 10.1111/j.2041-210x.2012.00251.x

[CIT0046] Schluter, D. (1996). Adaptive radiation along genetic lines of least resistance. Evolution, 50(5), 1766–1774. 10.1111/j.1558-5646.1996.tb03563.x 28565589

[CIT0047] Sgrò, C. M., & Hoffmann, A. A. (2004). Genetic correlations, tradeoffs and environmental variation. Heredity, 93(3), 241–248. 10.1038/sj.hdy.6800532 15280897

[CIT0048] Shaw, R. G., & Shaw, F. H. (2014). Quantitative genetic study of the adaptive process. Heredity, 112(1), 13–20. 10.1038/hdy.2013.42 23715015 PMC3860163

[CIT0049] Sheth, S. N., & Angert, A. L. (2018). Demographic compensation does not rescue populations at a trailing range edge. Proceedings of the National Academy of Sciences of the United States of America, 115(10), 2413–2418. 10.1073/pnas.1715899115 29463711 PMC5878003

[CIT0050] Steppan, S. J., Phillips, P. C., & Houle, D. (2002). Comparative quantitative genetics: Evolution of the G matrix. Trends in Ecology and Evolution, 17(7), 320–327. 10.1016/s0169-5347(02)02505-3

[CIT0051] van Heerwaarden, B., Kellermann, V., & Sgrò, C. M. (2016). Limited scope for plasticity to increase upper thermal limits. Functional Ecology, 30(12), 1947–1956. 10.1111/1365-2435.12687

[CIT0052] Via, S., Gomulkiewicz, R., de Jong, G., Scheiner, S. M., Schlichting, C. D., & Van Tienderen, P. H. (1995). Adaptive phenotypic plasticity: Consensus and controversy. Trends in Ecology and Evolution, 10, 212–217. 21237012 10.1016/s0169-5347(00)89061-8

[CIT0053] Walsh, B., & Blows, M. W. (2009). Abundant genetic variation plus strong selection = multivariate genetic constraints: A geometric view of adaptation. Annual Review of Ecology, Evolution, and Systematics, 40, 41–59.

[CIT0054] Walter, G. M. (2023). Experimental evidence that phenotypic evolution but not plasticity occurs along genetic lines of least resistance in homogeneous environments. American Naturalist, 201(4), E70–E89. 10.1086/723394 36957997

[CIT0055] Walter, G. M., Abbott, R. J., Brennan, A. C., Bridle, J. R., Chapman, M. A., Clark, J., Filatov, D., Nevado, B., Ortiz-Barrientos, D., & Hiscock, S. J. (2020a). *Senecio* as a model system for integrating studies of genotype, phenotype and fitness. New Phytologist, 226, 326–344. 31951018 10.1111/nph.16434

[CIT0056] Walter, G. M., Aguirre, J. D., Blows, M. W., & Ortiz-Barrientos, D. (2018). Evolution of genetic variance during adaptive radiation. American Naturalist, 191(4), E108–E128. 10.1086/696123 29570402

[CIT0057] Walter, G. M., Aguirre, J. D., Wilkinson, M. J., Richards, T. J., Blows, M. W., & Ortiz-Barrientos, D. (2020b). Loss of ecologically important genetic variation in late generation hybrids reveals links between adaptation and speciation. Evolution Letters, 4, 302–316. 32774880 10.1002/evl3.187 PMC7403682

[CIT0058] Walter, G. M., Clark, J., Cristaudo, A., Nevado, B., Catara, S., Paunov, M., Velikova, V., Filatov, D., Cozzolino, S., Hiscock, S. J., & Bridle, J. R. (2022a). Adaptive divergence generates distinct plastic responses in two closely related *Senecio* species. Evolution, 76, 1229–1245. 35344205 10.1111/evo.14478 PMC9322604

[CIT0059] Walter, G. M., Clark, J., Terranova, D., Cozzolino, S., Cristaudo, A., Hiscock, S. J., & Bridle, J. R. (2023). Hidden genetic variation in plasticity increases the potential to adapt to novel environments. New Phytologist, 239, 374–387. 36651081 10.1111/nph.18744

[CIT0060] Walter, G. M., Terranova, D., la Spina, E., Majorana, M. G., Pepe, G., du Plessis, S., Clark, J., Cozzolino, S., Cristaudo, A., Hiscock, S. J., & Bridle, J. R. (2022b). Adaptive plasticity in development rate and genetic variance in survival increase the potential for adapting to novel environments. bioRxiv 429835. 10.1101/2021.02.04.429835, preprint: not peer reviewed.

[CIT0061] Weis, A. E. (2018). Detecting the “invisible fraction” bias in resurrection experiments. Evolutionary Applications, 11(1), 88–95. 10.1111/eva.12533 29302274 PMC5748523

[CIT0062] Wood, C. W., & Brodie, E. D. III. (2015). Environmental effects on the structure of the G-matrix. Evolution, 69, 2927–2940. 26462609 10.1111/evo.12795

[CIT0063] Zu, P., Schiestl, F. P., Gervasi, D., Li, X., Runcie, D., & Guillaume, F. (2020). Floral signals evolve in a predictable way under artificial and pollinator selection in *Brassica rapa*. BMC Evolutionary Biology, 20(1), 127. 10.1186/s12862-020-01692-7 32972368 PMC7517814

