# Supplementary material for: Environmental effects on genetic variance are likely to constrain adaptation in novel environments
Source: Evol Lett. 2024 Jan 18;8(3):374–86. doi: 10.1093/evlett/qrad065 (PMC11285158; doi:10.1093/evlett/qrad065)
Supplement: qrad065_suppl_Supplementary_Material [file qrad065_suppl_supplementary_material.pdf]

Supplementary material for

Environmental effects on genetic variance are likely to constrain adaptation in novel environments

Greg M. Walter[*], *Keyne Monro, Delia Terranova, Enrico la Spina, Maria Majorana, Giuseppe Pepe, James Clark, Salvatore Cozzolino, Antonia Cristaudo, Simon J. Hiscock and Jon Bridle*

* Corresponding Author: Greg M. Walter

Email: greg.walter@monash.edu

Contents

Table S1 Sampling locations

Fig. S1 Map of sampling locations

Fig. S2 Demography during the field experiment

Table S2 Numbers of individuals that were measured in the field experiment

Fig. S3 Description of the covariance tensor approach

Fig. S4 Elevational change in mean for all five leaf traits.

Fig. S5 Significance test for estimates of genetic variance for each trait

Fig. S6 Posterior distribution of genetic variance for each trait

Table S3 G-matrices for all elevations and for both species

Fig. S7 Significance test for eigenvectors of **G**

Fig. S8 Significant eigentensors

Table S4 Summary of the covariance tensor analysis

Table S5 Coefficients for estimates of selection

Fig. S9 Visualising the selection gradients

Methods S1 Visualizing differences in mean multivariate phenotype

Methods S2 Comparing approaches for estimating genetic variance

Methods S3 Survival of families before and after measuring leaf phenotypes

Methods S4 Estimation of genetic variance before and after selection

Table S1: Location of sampled individuals for the parental generation of the breeding design for each species. The final two column denote the number of individuals used as sires and dams in the breeding design.

Species	Site	Elevation	Latitude	Longitude	# Sires	# Dams
S. aethn.	Etna South	2,600m	37°43'13.28"N	15° 0'3.54"E	1	1
		2,500m	37°43'3.80"N	14°59'59.20"E	8	7
		2,400m	37°42'46.50"N	14°59'41.30"E	5	5
		2,200m	37°42'24.82"N	14°59'42.69"E	2	5
	Etna North	2,600m	37°46'39.90"N	15° 0'23.00"E	8	4
		2,500m	37°46'53.70"N	15° 0'28.80"E	3	2
		2,400m	37°47'7.46"N	15° 0'35.05"E	4	8
		2,200m	37°47'32.82"N	15° 1'14.53"E	5	3
	**Totals**				**36**	**35**
S. chrys.	Bonnano	790m	37°38'24.92"N	15° 2'50.80"E	9	11
	Cacciola	680m	37°37'31.32"N	15° 3'26.71"E	7	6
	Poggofelice	526m	37°39'44.31"N	15° 5'48.55"E	6	6
	Spina	730m	37°39'19.27"N	15° 4'30.92"E	10	10
	Trecastagni	571m	37°36'46.67"N	15° 4'29.64"E	6	5
	**Totals**				**38**	**38**

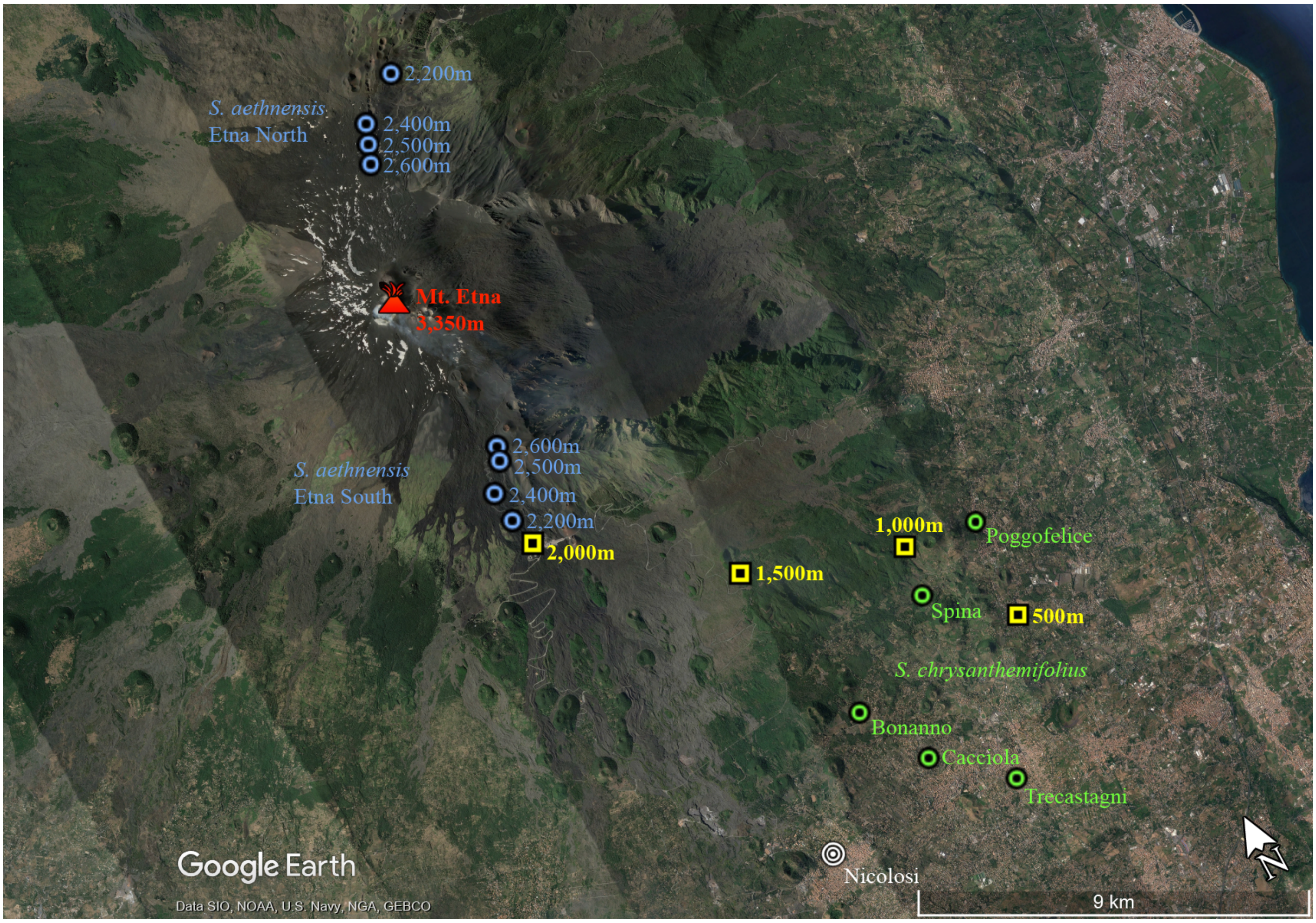

Fig. S1 Map with the locations of transplant sites (yellow), and the sites that the parental genotypes were sampled from for both *S. aethnensis* (blue) and *S. chrysanthemifolius* (green).

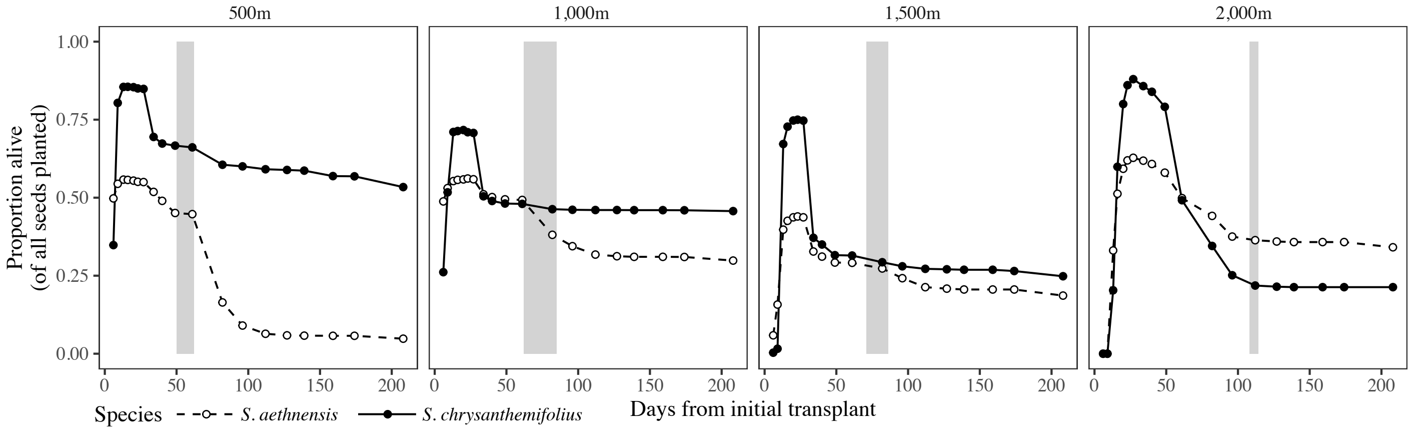

Fig. S2 Proportion of plants alive at each census day, for each transplant elevation. Filled circles and solid lines represent *S. chrysanthemifolius*, while unfilled circles and broken lines represent *S. aethnensis*. Grey bars denote the time period during which leaf measurements were taken for each elevation.

Table S2: The number of seedlings measured for leaf traits at each elevation and for each species.

Species	Elevation	Number of seedlings emerged	Number of seedlings measured	Proportion of plants that were measured	Average number of individuals measured per family (± 1 SD)
S. aethn.	500m	1224	689	0.56	7.33 (3.9)
	1,000m	1215	786	0.65	8.36 (3.8)
	1,500m	990	442	0.45	4.70 (3.1)
	2,000m	1393	644	0.46	6.85 (3.5)
	**Totals**	**4822**	**2561**	**0.53**	
S. chrys.	500m	2331	1683	0.72	15.58 (3.4)
	1,000m	1912	1144	0.60	10.59 (3.4)
	1,500m	2061	589	0.29	5.45 (2.3)
	2,000m	2414	482	0.20	4.46 (2.2)
	**Totals**	**8718**	**3898**	**0.45**	

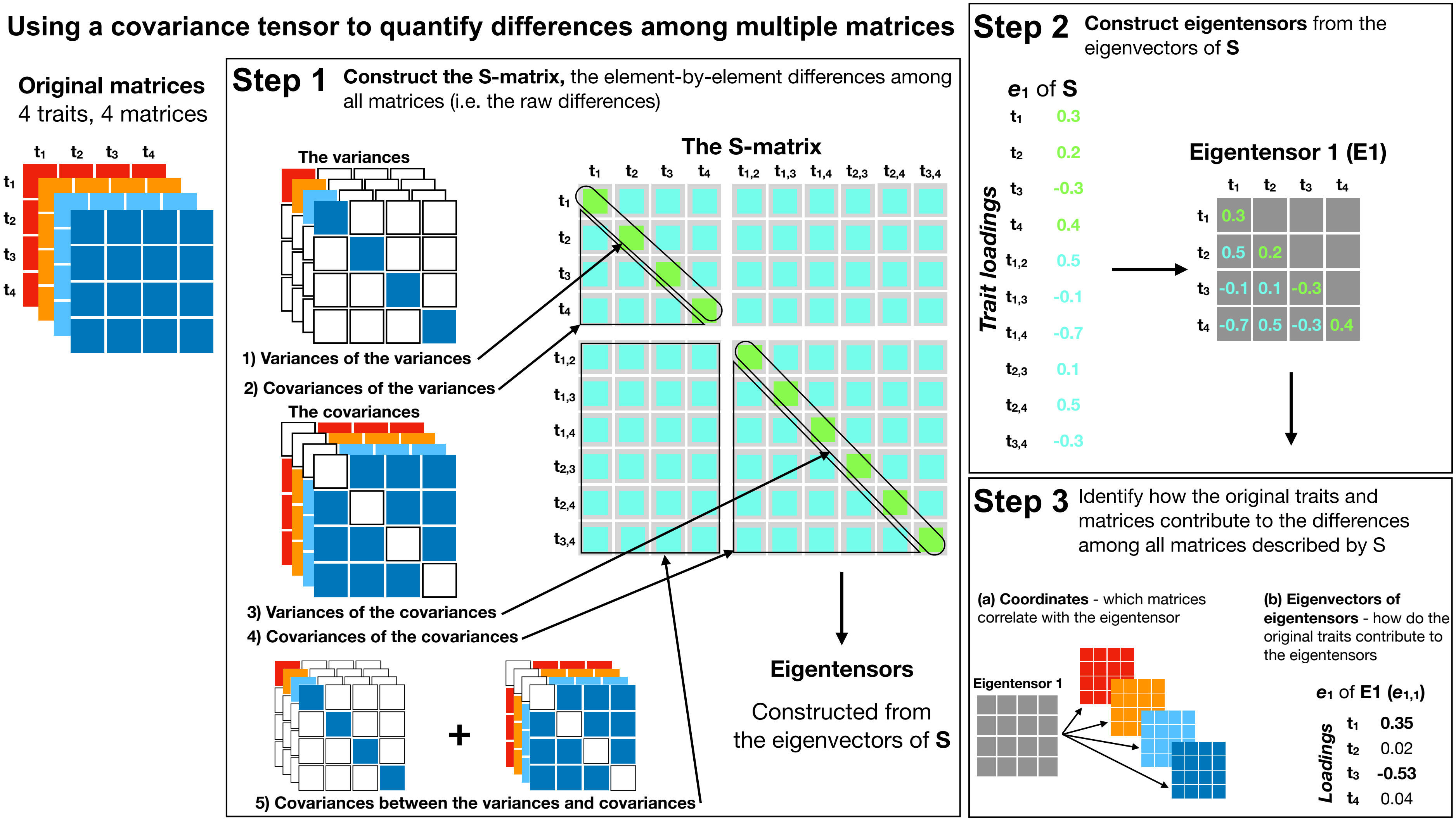

Fig. S3 Summary of the covariance tensor approach for comparing multiple matrices.

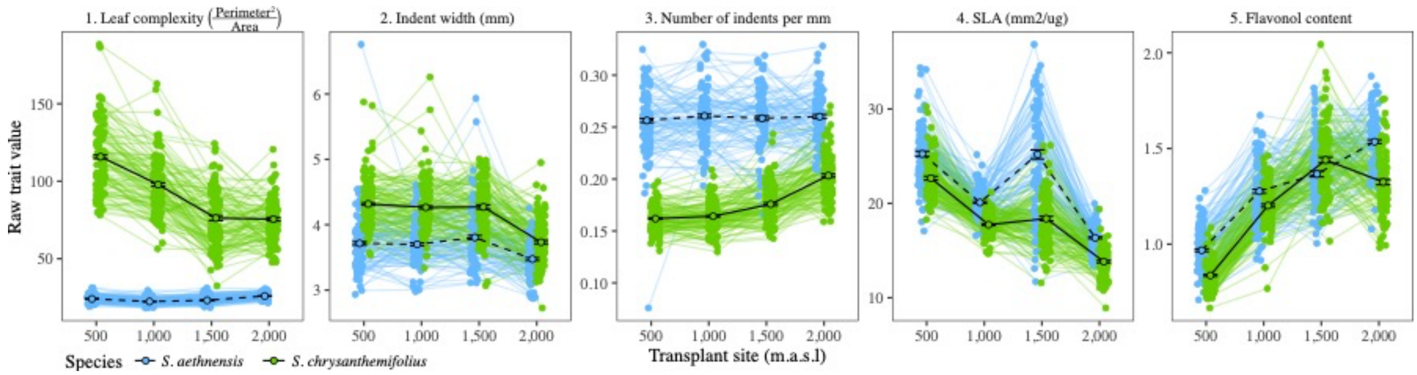

Fig. S4 Change in univariate traits across elevation for both species. *Senecio aethnensis* is represented in blue, and *S. chrysanthemifolius* in green. Black circles and lines represent the overall mean for each species at each elevation (confidence intervals represent one standard error). Coloured lines represent the mean for each full-sibling family at each elevation.

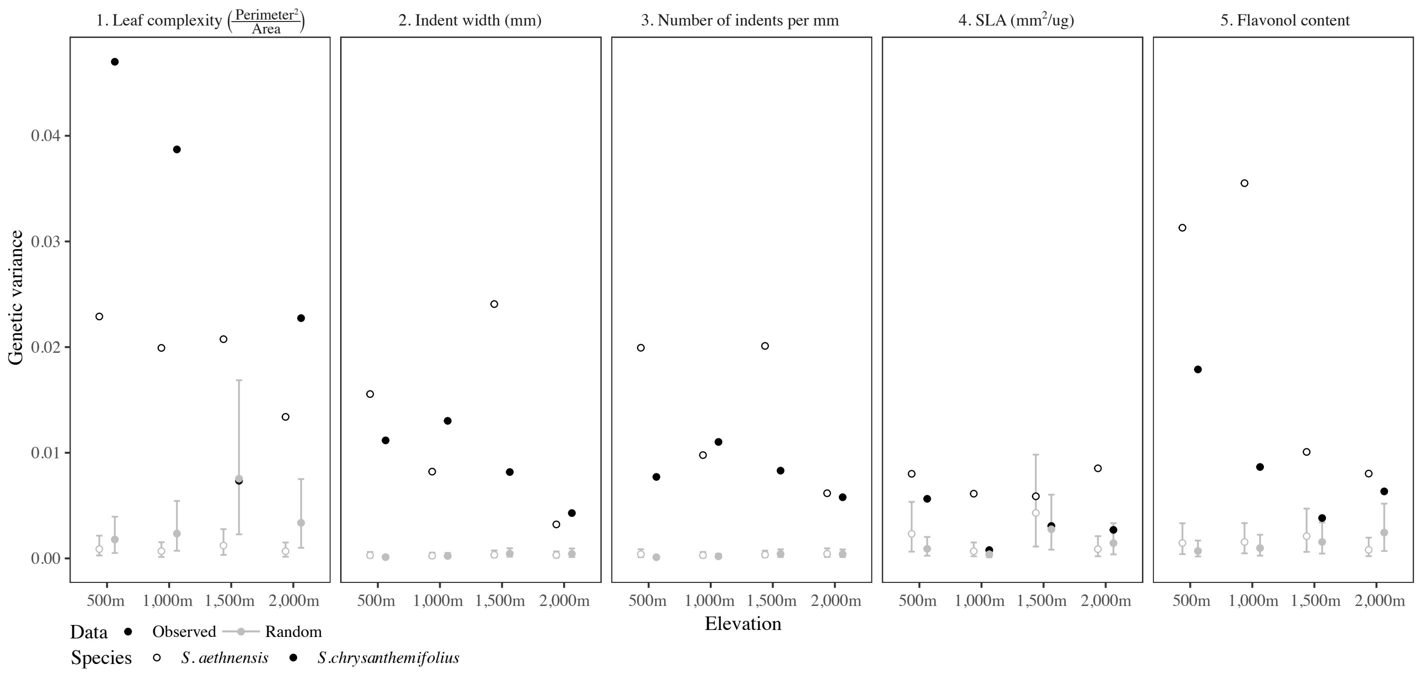

Fig. S5 Observed estimates of genetic variance (black circles) are greater than the null distribution (grey circles and 95% HPD interval) for most traits. Open circles represent *S. aethensis,* and closed circles *S. chrysanthemifolius.* Observed estimates represent the posterior mean of the observed models, while the random distribution is the distribution of the 1,000 models, each conducted on a randomisation of the pedigree and taking the mean from each model. Leaf complexity at 1,500m and SLA estimated at higher elevations for *S. chrysanthemifolius* were the only traits that showed less observed genetic variance than expected under random sampling.

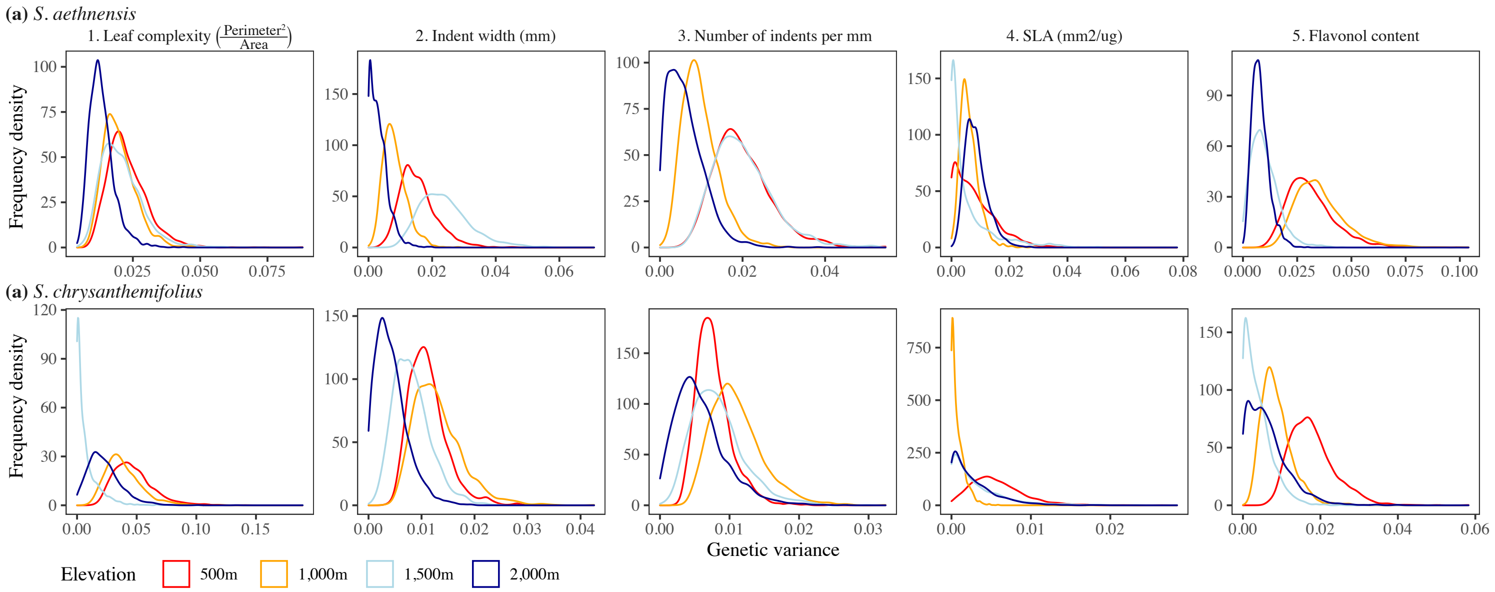

Fig. S6 Posterior distributions for estimates of genetic variance for each trait and species. Colours represent the different elevations (red=500m, orange=1,000m, light blue=1,500m and dark blue=2,000m). While the estimates of genetic variance for some traits are bounded by zero, genetic variance for most traits are normally distributed.

Table S3 G-matrices estimated at each elevation for **(a)** *S. aethnensis* and **(b)** *S. chrysanthemifolius*. Grey shading denotes the genetic variances along the diagonal. Genetic covariances are presented above the diagonal, and genetic correlations below the diagonal. Numbers in parentheses represent the 95% HPD interval for each parameter estimated. Numbers in bold denote genetic correlations with an absolute magnitude greater than 0.2 to aide interpretation. Traits: LC=leaf complexity, NI=number of leaf indents, IW=indent width, SLA=specific leaf area, and FL=flavonol content.

(a) *S. aethnensis*

	LC	IW	NI	SLA	FL
			**500m**		
LC	0.023<br>(0.011, 0.033)	0.003<br>(-0.004, 0.011)	0.000<br>(-0.008, 0.007)	-0.001<br>(-0.006, 0.005)	-0.005<br>(-0.015, 0.005)
IW	0.18<br>(-0.16, 0.53)	0.016<br>(0.007, 0.024)	-0.016<br>(-0.025, -0.007)	0.000<br>(-0.006, 0.006)	-0.012<br>(-0.022, -0.003)
NI	0.00<br>(-0.36, 0.36)	**-0.92**<br>**(-0.98, -0.87)**	0.02<br>(0.008, 0.03)	0.000<br>(-0.006, 0.007)	0.012<br>(0.003, 0.023)
SLA	-0.06<br>(-0.53, 0.42)	0.01<br>(-0.68, 0.77)	0.00<br>(-0.75, 0.67)	0.008<br>(0, 0.018)	-0.002<br>(-0.011, 0.005)
FL	**-0.20**<br>**(-0.52, 0.14)**	**-0.54**<br>**(-0.86, -0.25)**	**0.50**<br>**(0.19, 0.81)**	-0.14<br>(-0.72, 0.38)	0.031<br>(0.014, 0.047)
			**1,000m**		
LC	0.02<br>(0.01, 0.029)	0.002<br>(-0.003, 0.007)	-0.002<br>(-0.007, 0.004)	0.000<br>(-0.005, 0.004)	-0.010<br>(-0.019, -0.001)
IW	0.13<br>(-0.22, 0.51)	0.008<br>(0.003, 0.014)	-0.008<br>(-0.014, -0.002)	0.001<br>(-0.002, 0.004)	-0.009<br>(-0.016, -0.001)
NI	-0.12<br>(-0.49, 0.23)	**-0.94**<br>**(-1, -0.88)**	0.01<br>(0.003, 0.016)	-0.001<br>(-0.005, 0.003)	0.009<br>(0.001, 0.017)
SLA	-0.02<br>(-0.38, 0.37)	0.18<br>(-0.33, 0.67)	-0.17<br>(-0.72, 0.29)	0.006<br>(0.001, 0.011)	-0.007<br>(-0.014, -0.001)
FL	**-0.38**<br>**(-0.69, -0.11)**	**-0.51**<br>**(-0.83, -0.18)**	**0.51**<br>**(0.16, 0.81)**	**-0.47**<br>**(-0.78, -0.14)**	0.036<br>(0.018, 0.052)
			**1,500m**		
LC	0.021<br>(0.009, 0.031)	0.008<br>(-0.001, 0.016)	-0.008<br>(-0.017, 0)	0.000<br>(-0.006, 0.006)	-0.005<br>(-0.011, 0.002)
IW	**0.33**<br>**(-0.02, 0.65)**	0.024<br>(0.012, 0.036)	-0.021<br>(-0.032, -0.01)	-0.001<br>(-0.011, 0.008)	-0.008<br>(-0.017, 0.001)
NI	**-0.40**<br>**(-0.73, -0.09)**	**-0.97**<br>**(-1, -0.94)**	0.02<br>(0.008, 0.03)	0.001<br>(-0.006, 0.011)	0.007<br>(-0.001, 0.016)
SLA	0.01<br>(-0.64, 0.63)	-0.09<br>(-0.99, 0.83)	0.10<br>(-0.84, 0.99)	0.006<br>(0, 0.017)	-0.001<br>(-0.006, 0.004)
FL	**-0.33**<br>**(-0.78, 0.05)**	**-0.54**<br>**(-0.99, -0.08)**	**0.54**<br>**(0.07, 1)**	-0.06<br>(-0.8, 0.76)	0.01<br>(0, 0.018)
			**2,000m**		
LC	0.013<br>(0.006, 0.02)	0.000<br>(-0.003, 0.003)	0.002<br>(-0.002, 0.006)	-0.001<br>(-0.005, 0.003)	-0.002<br>(-0.007, 0.002)
IW	-0.07<br>(-0.58, 0.45)	0.003<br>(0, 0.007)	-0.004<br>(-0.008, 0)	0.000<br>(-0.003, 0.003)	-0.001<br>(-0.004, 0.002)
NI	**0.28**<br>**(-0.11, 0.74)**	**-0.67**<br>**(-0.98, -0.18)**	0.006<br>(0, 0.012)	0.001<br>(-0.003, 0.004)	0.000<br>(-0.004, 0.004)
SLA	-0.11<br>(-0.52, 0.25)	-0.05<br>(-0.58, 0.51)	0.11<br>(-0.38, 0.63)	0.009<br>(0.003, 0.015)	-0.002<br>(-0.005, 0.002)
FL	-0.19<br>(-0.54, 0.19)	-0.14<br>(-0.73, 0.44)	-0.06<br>(-0.61, 0.49)	-0.18<br>(-0.62, 0.24)	0.008<br>(0.002, 0.014)

Table S3 (*continued*)

(b) *S. chrysanthemifolius*

	LC	IW	NI	SLA	FL
			**500m**		
LC	0.047 (0.023, 0.072)	0.000 (-0.009, 0.008)	-0.003 (-0.01, 0.004)	-0.002 (-0.009, 0.005)	-0.013 (-0.023, -0.002)
IW	0.01 (-0.35, 0.37)	0.011 (0.006, 0.016)	-0.008 (-0.012, -0.004)	-0.004 (-0.007, 0)	-0.002 (-0.007, 0.003)
NI	-0.17 (-0.54, 0.16)	**-0.87 (-0.97, -0.79)**	0.008 (0.004, 0.011)	0.002 (-0.001, 0.005)	0.003 (-0.001, 0.008)
SLA	-0.08 (-0.53, 0.32)	**-0.49 (-0.88, -0.12)**	**0.37 (-0.04, 0.8)**	0.006 (0, 0.01)	-0.002 (-0.007, 0.002)
FL	**-0.47 (-0.75, -0.18)**	-0.15 (-0.48, 0.18)	**0.26 (-0.06, 0.58)**	**-0.21 (-0.62, 0.21)**	0.018 (0.009, 0.027)
			**1,000m**		
LC	0.039 (0.016, 0.061)	-0.004 (-0.013, 0.006)	0.000 (-0.008, 0.008)	-0.001 (-0.005, 0.002)	-0.011 (-0.019, -0.003)
IW	-0.16 (-0.56, 0.21)	0.013 (0.006, 0.019)	-0.01 (-0.015, -0.004)	0.000 (-0.002, 0.002)	-0.002 (-0.007, 0.002)
NI	0.02 (-0.33, 0.45)	**-0.83 (-0.95, -0.71)**	0.011 (0.005, 0.016)	0.000 (-0.002, 0.002)	0.003 (-0.001, 0.007)
SLA	**-0.24 (-0.87, 0.32)**	0.00 (-0.66, 0.77)	-0.06 (-0.86, 0.57)	0.001 (0, 0.002)	0.000 (-0.001, 0.002)
FL	**-0.61 (-0.89, -0.32)**	**-0.21 (-0.61, 0.2)**	**0.29 (-0.07, 0.69)**	0.13 (-0.46, 0.82)	0.009 (0.002, 0.014)
			**1,500m**		
LC	0.007 (0, 0.02)	-0.001 (-0.008, 0.004)	0.000 (-0.006, 0.006)	0.000 (-0.003, 0.004)	-0.001 (-0.004, 0.003)
IW	-0.13 (-0.95, 0.67)	0.008 (0.003, 0.014)	-0.007 (-0.012, -0.002)	-0.002 (-0.005, 0.002)	-0.001 (-0.004, 0.002)
NI	0.01 (-0.75, 0.9)	**-0.79 (-0.96, -0.64)**	0.008 (0.003, 0.014)	0.001 (-0.002, 0.005)	0.001 (-0.003, 0.004)
SLA	0.06 (-0.74, 0.79)	**-0.35 (-0.98, 0.38)**	**0.29 (-0.48, 0.92)**	0.003 (0, 0.007)	0.000 (-0.002, 0.002)
FL	-0.08 (-0.82, 0.6)	**-0.23 (-0.9, 0.43)**	0.18 (-0.42, 0.91)	0.05 (-0.64, 0.76)	0.004 (0, 0.009)
			**2,000m**		
LC	0.023 (0, 0.041)	-0.002 (-0.008, 0.004)	-0.003 (-0.011, 0.004)	0.001 (-0.004, 0.006)	-0.004 (-0.012, 0.003)
IW	**-0.22 (-0.86, 0.38)**	0.004 (0, 0.008)	-0.003 (-0.007, 0.001)	-0.001 (-0.003, 0.001)	0.002 (-0.001, 0.005)
NI	**-0.22 (-0.8, 0.36)**	**-0.49 (-0.92, 0.03)**	0.006 (0, 0.011)	0.000 (-0.002, 0.003)	-0.001 (-0.004, 0.003)
SLA	0.12 (-0.51, 0.81)	**-0.30 (-0.95, 0.31)**	0.08 (-0.59, 0.79)	0.003 (0, 0.007)	-0.001 (-0.004, 0.001)
FL	**-0.33 (-0.86, 0.23)**	**0.39 (-0.18, 0.95)**	-0.12 (-0.8, 0.49)	**-0.23 (-0.85, 0.43)**	0.006 (0, 0.013)

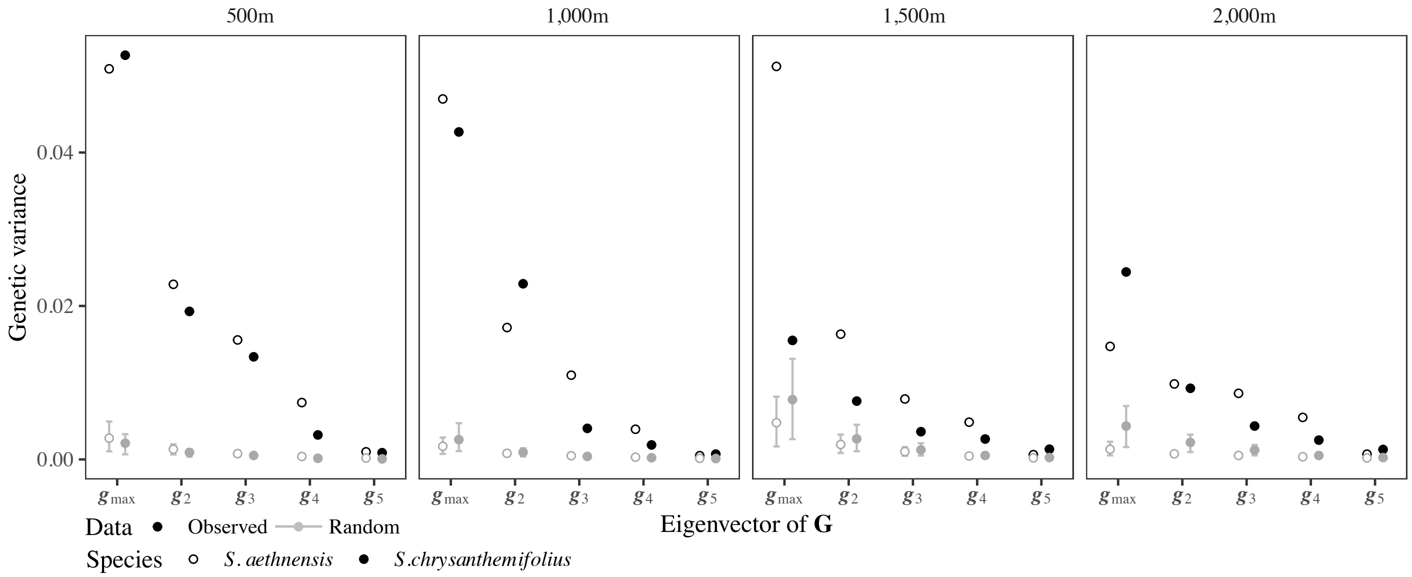

Fig. S7 The first three eigenvectors of observed **G** (black circles) that describe >90% of total genetic variance also captured more genetic variance than expected under random sampling (grey circles, credible intervals and grey shading). Open circles represent *S. aethnensis,* and closed circles represent *S. chrysanthemifolius*. Credible intervals represent the 95% HPD (Highest Posterior Density) intervals for the models applied to the 1,000 randomisations of the data.

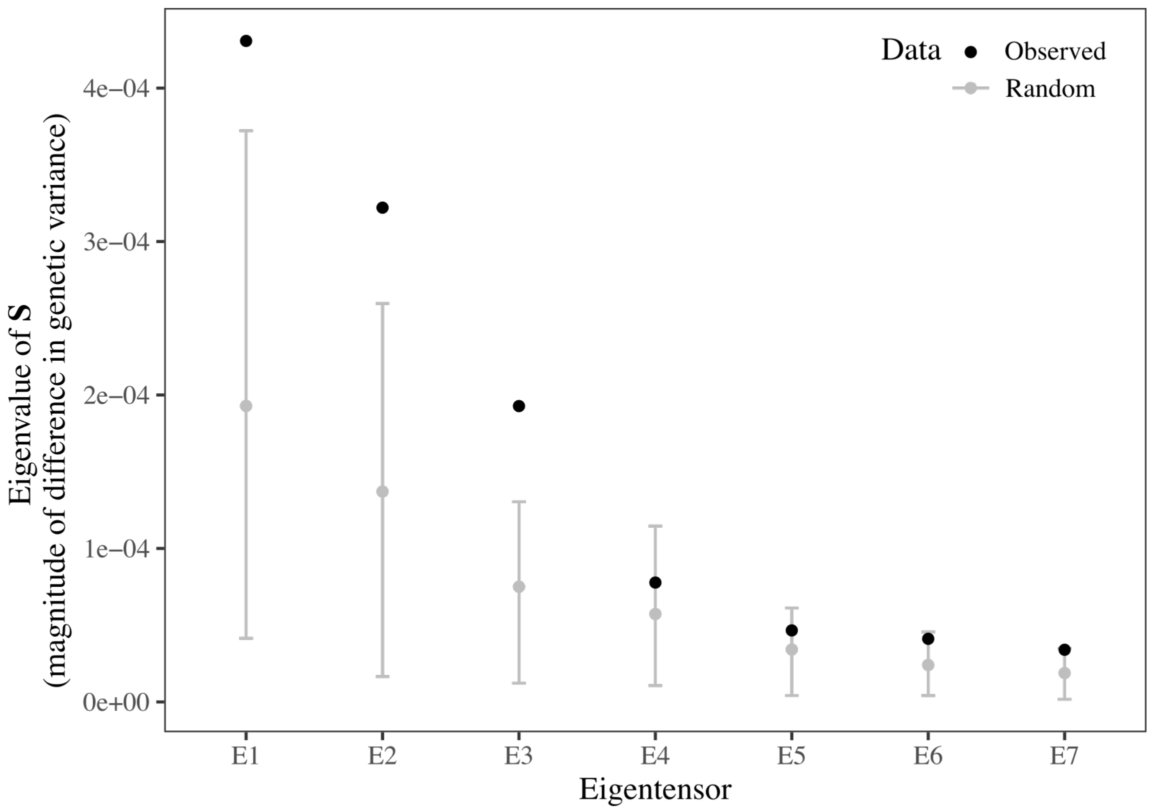

Fig. S8 Comparing the magnitude of difference in genetic variance captured by the tensor applied to the observed estimates of genetic variance (black circles) versus the null distribution (grey). The grey credible intervals represent the 95% HPD interval for the null distribution, which is calculated by re-applying equation 1 to data containing no differences in genetic variance and taking the mean from each implementation. The first three eigentensors describe greater differences in genetic variance than expected under the null distribution.

Table S4 Summary of the covariance tensor analysis that captured differences in **G** across elevation for both species. Presented are the 7 non-zero eigentensors. Eigenvalue of **S** represents the amount of difference in genetic variance described by each eigentensor, with 'Prop. **S**' representing the proportion of the total difference in genetic variance. λ represents the amount of difference in genetic variance described by each eigenvector of eigentensor, with 'Prop λ' representing the proportion that each eigenvector contributes to describing the difference in genetic variance described by the corresponding eigentensor. HPD represents the 90% Highest Posterior Density intervals.

**Eigen-tensor**	**Eigenvalue of S (HPD)**	**Prop. S**	**Eigenvector of eigentensor**	**Prop. λ**	**λ**	**P2A**	**IW**	**Nind**	**SLA**	**Flav**
E1	0.000431	0.35	$\boldsymbol{e}_{1,1}$	0.66	-0.95	0.40	0.47	-0.46	0.05	-0.63
	(0.000142,		$\boldsymbol{e}_{1,2}$	0.19	-0.27	0.91	-0.22	0.28	-0.12	0.19
	0.000729)		$\boldsymbol{e}_{1,3}$	0.12	-0.17	-0.08	-0.51	0.41	0.19	-0.72
			$\boldsymbol{e}_{1,4}$	0.02	-0.03	0.10	0.09	0.03	0.97	0.19
			$\boldsymbol{e}_{1,5}$	0.01	0.01	0.06	-0.68	-0.73	0.07	0.07
E2	0.000322	0.27	$\boldsymbol{e}_{2,1}$	0.61	0.89	0.96	-0.15	0.00	-0.03	-0.23
	(6.33e-05,		$\boldsymbol{e}_{2,2}$	0.30	-0.44	-0.03	0.57	-0.62	0.13	-0.53
	0.000598)		$\boldsymbol{e}_{2,3}$	0.04	-0.06	-0.19	-0.25	0.08	-0.78	-0.54
			$\boldsymbol{e}_{2,4}$	0.04	-0.05	0.15	0.12	-0.54	-0.55	0.61
			$\boldsymbol{e}_{2,5}$	0.01	0.01	0.13	0.76	0.57	-0.27	0.08
E3	0.000193	0.16	$\boldsymbol{e}_{3,1}$	0.45	-0.71	-0.10	-0.04	0.06	-0.24	0.96
	(3.3e-05,		$\boldsymbol{e}_{3,2}$	0.43	0.69	-0.35	-0.69	0.62	0.11	-0.08
	0.000332)		$\boldsymbol{e}_{3,3}$	0.07	0.11	0.82	-0.53	-0.14	0.13	0.11
			$\boldsymbol{e}_{3,4}$	0.04	-0.06	-0.26	-0.05	-0.33	0.88	0.21
			$\boldsymbol{e}_{3,5}$	0.01	-0.02	0.36	0.49	0.70	0.37	0.10
E4	7.78e-05	0.06	$\boldsymbol{e}_{4,1}$	0.63	0.90	0.63	-0.54	0.55	-0.04	0.13
	(6.86e-06,		$\boldsymbol{e}_{4,2}$	0.30	-0.43	0.77	0.40	-0.46	-0.08	-0.15
	0.000149)		$\boldsymbol{e}_{4,3}$	0.04	-0.06	-0.05	0.08	-0.06	-0.82	0.57
			$\boldsymbol{e}_{4,4}$	0.02	0.03	-0.08	-0.48	-0.26	-0.49	-0.67
			$\boldsymbol{e}_{4,5}$	0.01	0.01	0.01	-0.56	-0.65	0.29	0.43
E5	4.66e-05	0.04	$\boldsymbol{e}_{5,1}$	0.59	-0.92	0.17	0.41	-0.40	-0.66	0.46
	(4.65e-06,		$\boldsymbol{e}_{5,2}$	0.17	-0.27	0.18	0.00	-0.01	0.60	0.78
	9.02e-05)		$\boldsymbol{e}_{5,3}$	0.17	0.26	0.38	0.44	-0.56	0.42	-0.41
			$\boldsymbol{e}_{5,4}$	0.05	-0.07	0.77	0.14	0.60	-0.13	-0.07
			$\boldsymbol{e}_{5,5}$	0.03	0.04	0.44	-0.79	-0.41	-0.13	-0.01
E6	4.11e-05	0.03	$\boldsymbol{e}_{6,1}$	0.45	0.76	0.78	-0.05	0.10	-0.32	0.53
	(5.61e-06,		$\boldsymbol{e}_{6,2}$	0.36	-0.61	0.45	-0.49	0.31	0.15	-0.66
	7.56e-05)		$\boldsymbol{e}_{6,3}$	0.12	0.21	-0.29	-0.18	0.19	-0.90	-0.18
			$\boldsymbol{e}_{6,4}$	0.06	-0.10	0.33	0.75	-0.23	-0.20	-0.49
			$\boldsymbol{e}_{6,5}$	0.02	0.03	0.09	-0.41	-0.89	-0.12	-0.09
E7	3.4e-05	0.03	$\boldsymbol{e}_{7,1}$	0.45	-0.78	0.29	0.29	-0.30	0.82	-0.28
	(1.9e-06,		$\boldsymbol{e}_{7,2}$	0.33	0.56	0.15	0.32	-0.34	-0.53	-0.69
	6.6e-05)		$\boldsymbol{e}_{7,3}$	0.16	-0.27	-0.12	0.62	-0.46	-0.14	0.61
			$\boldsymbol{e}_{7,4}$	0.04	0.07	0.72	0.37	0.56	-0.13	0.16
			$\boldsymbol{e}_{7,5}$	0.03	-0.04	0.60	-0.53	-0.52	-0.13	0.24

Table S5 Estimates of selection coefficients for the phenotypic selection gradients (***β***) estimated for *S. aethnensis* at each elevation away from its home site.

Elevation	Fixed effect	Coefficient	Df	Sum of Squares	Pr(>Chi)	95% Confidence interval
**500m**	Intercept	-6.51				-11.89, -1.29
	**Complexity**	**3.52**	**1**	**31.223**	**<0.001**	**2.27, 4.81**
	Indent width	0.2	1	0.021	0.884	-2.44, 2.83
	Number indents	-0.01	1	0	0.991	-2.42, 2.42
	SLA	-0.27	1	0.587	0.444	-0.98, 0.42
	**Flavonol**	**1.56**	**1**	**10.272**	**0.001**	**0.61, 2.53**
**1,000m**	Intercept	-3.65				-8.61, 1.31
	**Complexity**	**1.9**	**1**	**7.942**	**0.005**	**0.57, 3.28**
	Indent width	-0.47	1	0.152	0.697	-2.8, 1.92
	Number indents	0.6	1	0.288	0.591	-1.6, 2.79
	**SLA**	**2.58**	**1**	**14.042**	**<0.001**	**1.21, 4.01**
	Flavonol	0.59	1	1.763	0.184	-0.28, 1.48
**1,500m**	Intercept	-2.61				-11.13, 5.71
	Complexity	1.03	1	1.052	0.305	-0.92, 3.09
	Indent width	-0.44	1	0.042	0.837	-4.5, 3.82
	Number indents	0.13	1	0.004	0.949	-3.9, 4.19
	**SLA**	**1.22**	**1**	**7.357**	**0.007**	**0.33, 2.18**
	**Flavonol**	**2.5**	**1**	**11.585**	**0.001**	**1.05, 3.99**

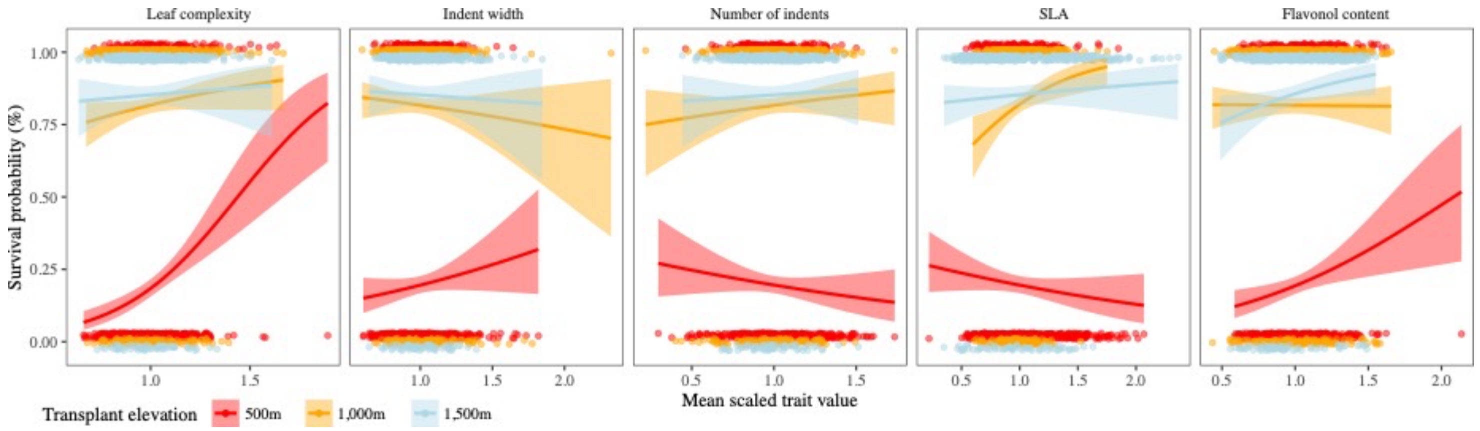

Fig. S9 Visualising the selection gradients calculated (from **Table S5**) for the five leaf traits (each panel), only using data collected for *S. aethnensis* at 500-1,500m. Regressions are calculated using survival as a binary trait with the shaded ribbons representing one standard error.

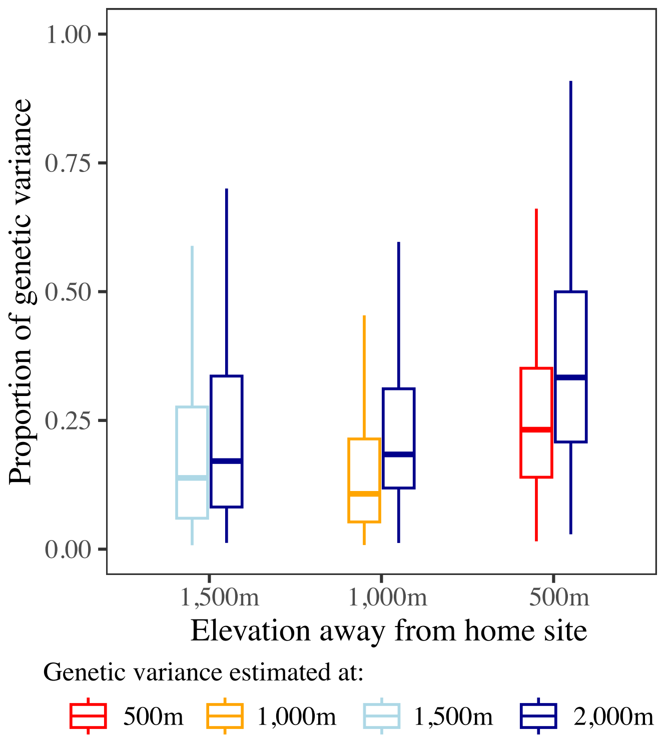

Fig. S10 Projection of genotypic selection gradients, which were calculated with the same traits and used the mean of each full-sibling family to quantify genotypic selection on each trait. Results are similar to those presented in text for the phenotypic selection gradients.

Methods S1 – Estimation of the D-matrix

To visualise how the two species differed across elevation we first constructed a D-matrix, the covariance matrix representing differences in mean multivariate phenotype between species and across elevation. To construct **D**, from the MANOVA we extracted the Sums of Squares and Cross-Product (SSCP) matrices for each fixed effect ($SSCP_S$ = species; $SSCP_E$ = elevation; $SSCP_{S\times E}$ = species×elevation) and the error term ($SSCP_R$). We then estimated $SSCP_H$ ($SSCP_H = SSCP_S + SSCP_E + SSCP_{S\times E}$), which calculates the difference in mean across all elevations for both species. We calculated Mean Square (MS) matrices by dividing the SSCP matrices by their corresponding degrees of freedom ($MS_H = \frac{SSCP_H}{7}$; $MS_R = \frac{SSCP_R}{6{,}446}$). We then estimated **D** using

$$\mathbf{D} = \frac{MS_H - MS_R}{nf}, \qquad (1)$$

where *nf* represents the average number of individuals measured for each species at each elevation, calculated from equation 9 in Martin et al. (2008). We used the eigenvectors of **D** to visualise differences in multivariate phenotype across elevation for both species.

Methods S2 – Comparing approaches for estimating genetic variance

To ensure that analysing our data using a nested paternal half-sibling approach is appropriate, we compared estimates of genetic variance of our leaf traits when analysing our design as a paternal half-sibling (main text; equation 2 below) versus a fully factorial North Carolina II (NCII) design (equation 3 below). The NCII design involves reciprocally crossing sires and dams in blocks, such that all sires and dams are mated to each other within each block. This means that sires are crossed to multiple dams and dams to multiple sires, which differs to the paternal half-sibling design where dams are nested within sires. The benefit of an NCII is that it is possible to look at variance due to dominance and epistasis by estimating variance among full-sibling families by estimating the sire×dam interaction. See Lynch and Walsh (1998) for further details.

Both approaches were applied to our leaf data using *MCMCglmm* as described in the main text. The paternal half-sibling design (repeated from the main text) is

$$\boldsymbol{y_{ijkl}} = s_i + d_{j(i)} + b_k + e_{l(ijk)}\,, \qquad (2)$$

where leaf traits are the multivariate response variable ($\boldsymbol{y_{ijkl}}$), s_i is the *i*th sire, $d_{j(i)}$ the *j*th dam nested within sire and $e_{l(ijk)}$ is the residual. Analysis using an NCII design is

$$\boldsymbol{y_{ijkl}} = s_i + d_j + s_i \times d_j + b_k + e_{l(ijk)}\,, \qquad (3)$$

where the additional $s_i \times d_j$ component captures the variance among full-sibling families, which is due to epistasis and dominance. Both approaches are expected to produce the same estimates of genetic variance because when analysing an NCII design with a paternal half-sibling approach, removing the sire×dam interaction will move the variance into the residual and not over-inflate the estimates of genetic variance Schielzeth and Nakagawa (2013). To test that both approaches produced the same estimates of **G**, we projected the eigenvectors of observed **G** used in the main text (i.e., estimated from the paternal half-sibling design) through the posterior distribution of observed **G** estimated using both approaches. **Fig. S11** shows that, as expected, eigenvectors of **G** described the same amount of genetic variance for both approaches, confirming the expectation detailed in Schielzeth and Nakagawa (2013) that when analysing full-factorial designs, estimating genetic variance with a paternal half-sibling is appropriate. Analysing **G** estimated using the NCII approach with the same analytical techniques as described in the main text (covariance tensor and matrix projections) produced the same results as presented in the manuscript (for simplicity, results not shown).

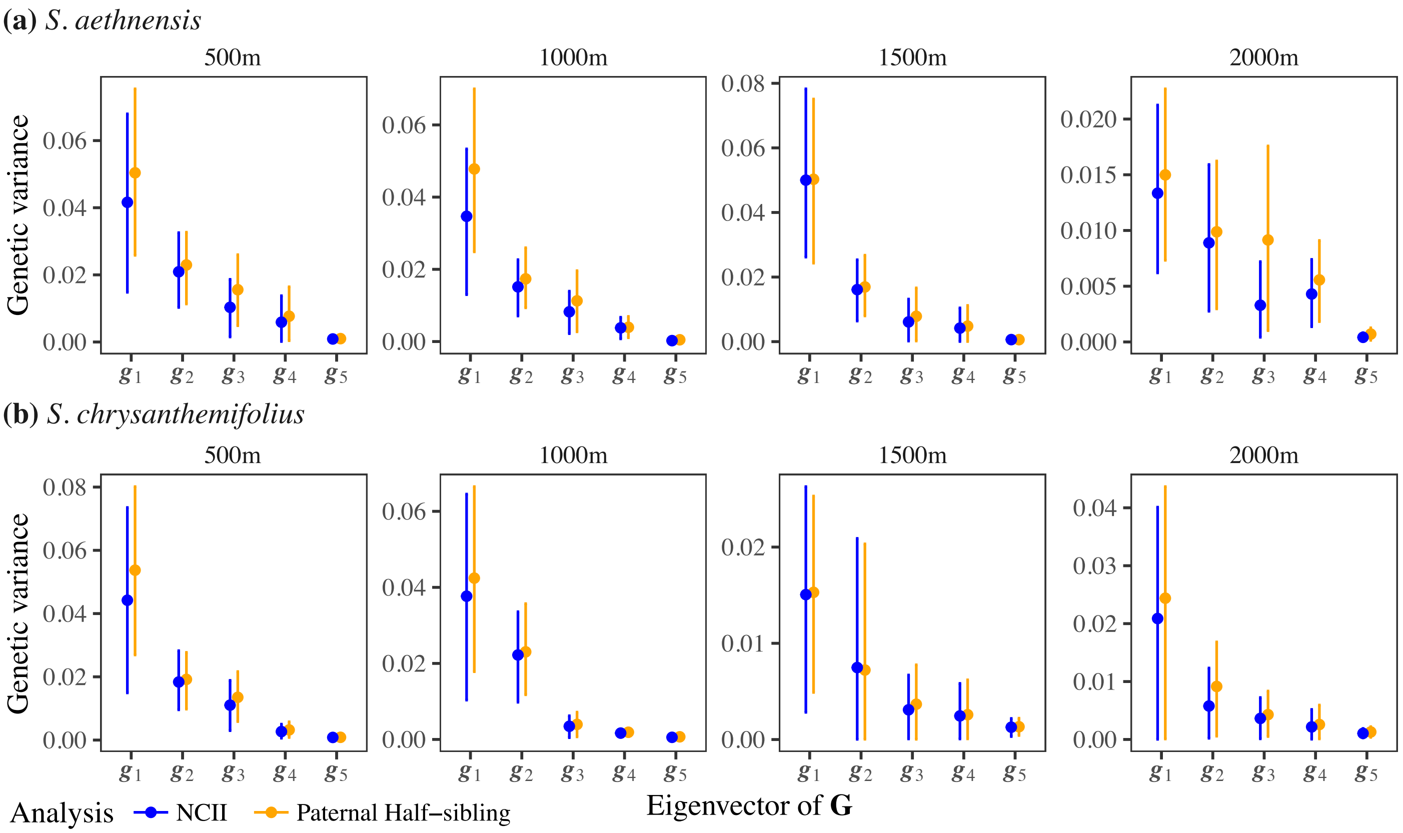

Fig. S11 No difference in **G** when estimated using an NCII (blue) versus a paternal half-sibling (orange) design. Genetic variance estimated for **(a)** *S. aethnensis* and **(b)** *S. chrysanthemifolius,* with each column representing a different transplant elevation. Circles and credible intervals represent the mean and 95% Highest Posterior Density interval for the projection of the eigenvectors of observed **G** through the posterior distribution of **G**.

Methods S3 – Survival of families before and after measuring leaf phenotypes

To check that early mortality would not bias our estimates of genetic variance, plasticity and selection, we tested whether families showed similar levels of mortality early in life history (pre-selection) compared to when measurements were taken. We therefore tested the interaction between family and time-point (pre-selection versus measurement point) using a generalised linear model with a binomial error distribution. The only other factor in the model was experimental block. We predicted that where the Family×time-point interaction is not significant there is no strong evidence for selection against certain families before leaf measurements were taken (i.e., mortality was effectively random).

As predicted, we found no significant Family×time-point interaction for either species at any of the transplant elevations (**Table S6**). Non-random selection should not have affected estimates of selection, genetic variance or plasticity in our experiment.

Table S6 Likelihood ratio tests from a type III ANOVA. Time-point represents mortality data taken at two time-points, the first at peak survival and the second when leaf measurements were taken.

Species	Elevation	Factor	χ^2	Df	P-value
**(a)** *S. aethnensis*	500m	Time-point	0.000	1	0.999
		Family	94.262	93	0.444
		Block	151.044	4	<0.001
		Time-point × Family	50.740	93	1
	1,000m	Time-point	2825.142	1	<0.001
		Family	103.127	93	0.222
		Block	228.536	4	<0.001
		Time-point × Family	67.126	93	0.98
	1,500m	Time-point	0.000	1	0.999
		Family	96.927	93	0.37
		Block	54.595	4	<0.001
		Time-point × Family	84.184	93	0.732
	2,000m	Time-point	41872.689	1	<0.001
		Family	72.567	93	0.942
		Block	260.905	4	<0.001
		Time-point × Family	46.122	93	1
**(b)** *S. chrysanthemifolius*	500m	Time-point	45935.219	1	<0.001
		Family	87.994	107	0.91
		Block	241.100	4	<0.001
		Time-point × Family	70.887	107	0.997
	1,000m	Time-point	80049.200	1	<0.001
		Family	109.927	107	0.404
		Block	128.674	4	<0.001
		Time-point × Family	112.731	107	0.333
	1,500m	Time-point	1096.585	1	<0.001
		Family	102.286	107	0.611
		Block	259.717	4	<0.001
		Time-point × Family	80.504	107	0.974
	2,000m	Time-point	3656.939	1	<0.001
		Family	60.103	107	1
		Block	167.158	4	<0.001
		Time-point × Family	79.262	107	0.98

Methods S4 – Estimation of genetic variance when there is viability selection

Estimating genetic variance requires assumptions of no selection that is difficult uphold when measuring under more natural field conditions (Milner et al. 2000). To check that selection did not bias our estimates of genetic variance at novel elevations (the focus of the interpretation of the results), we conducted an additional analysis for each species.

Senecio aethnensis planted at 500m

We calculated genetic variance in the five leaf traits before and after selection for *S. aethnensis* at the 500m transplant elevation. We chose this species at this elevation because measurements were taken before selection at 500m (unlike at higher elevations), and the species native to the 500m elevation did not endure strong selection. Estimating the effect of selection on genetic variance for *S. aethnensis* experiencing a novel elevation provides an upper bound for the effect of selection on genetic variance. For pre-selection estimates of genetic variance, we used estimates provided in the main text. For post-selection estimates of genetic variance, we estimated genetic variance in the same way, but only for the individuals that were alive at the end of summer, which is when patterns of adaptive divergence emerge (**Fig. 2c**, main text) and *S. aethnensis* shows greater mortality at 500m than the native *S. chrysanthemifolius.* To compare pre- and post-selection estimates of genetic variance, we compared total genetic variance (i.e., the trace of **G**) and then projected the eigenvectors of observed **G** estimated for all individuals (pre-selection) through the posterior distribution for both estimates (pre- and post-selection) of **G**.

We found that total genetic variance was very similar when estimated pre- versus post-selection (**Fig. S12a**). Furthermore, eigenvectors of **G** (pre-selection) described the same amount of genetic variance in both pre- and post-selection estimates of **G** (**Fig. S12b**). These analyses therefore suggest that selection did not have a strong influence on the amount of genetic variance or affect the structure of **G**.

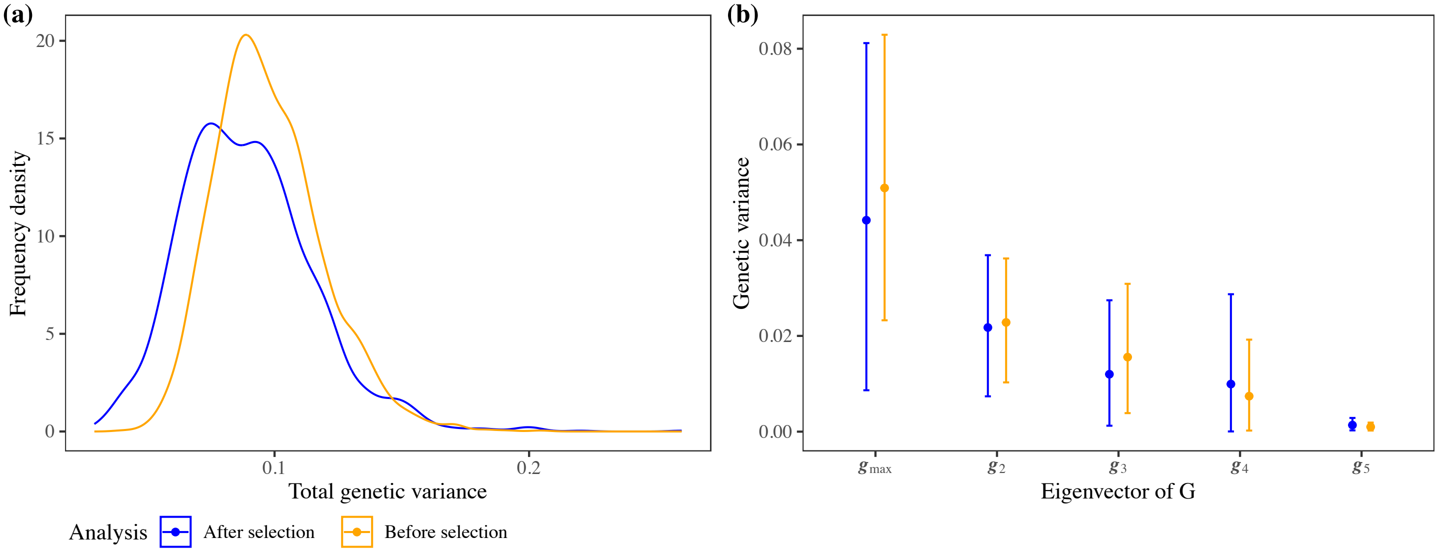

Fig. S12 Comparing genetic variance estimated before and after selection. **(a)** Estimates of total additive genetic variance are the same when estimated on individuals before selection (orange) versus surviving individuals after selection (blue). Density distributions represent the posterior distribution of the sum of the diagonal components of **G** (i.e., the trace) for each MCMC sample. **(b)** Eigenvectors of **G** (estimated before selection) describe the same amount of genetic variance in **G** estimated before and after selection. Credible intervals represent the 95% posterior distribution for the projection of each eigenvector for each MCMC sample of **G**.

Senecio chrysanthemifolius planted at 2,000m

In our current study (2019 experiment), individuals of *S. chrysanthemifolius* died before we could measure their leaves at higher elevations. This meant that we were unable to quantify genetic variance prior to selection in the current study. However, an experiment conducted in 2018 transplanted the same breeding design, but used offspring raised in the glasshouse and transplanted as cuttings (clones) in the field. Briefly, three seeds from each of the same 108 full-sibling families were propagated in the glasshouse (n=312 plants). When mature, we took cuttings from each plant (genotype) and then transplant eight cuttings of each genotype at each transplant elevation (500m, 1,500m and 2,000m). After four months, we took leaf samples and measured the same traits as presented in the current study. This design meant that we were able to bypass selection and measure replicate clones of each genotype at each elevation. Further details of the 2018 experiment are found in Walter et al. (2023).

Using the data from the 2018 experiment (where mortality did not affect the measurement of leaf traits), we estimated genetic variance for the same leaf traits at 500m, 1,500m and 2,000m. To estimate genetic variance in the same traits we used MCMCglmm to apply the linear mixed model

$$\boldsymbol{y_{ijklm}} = s_i + d_{j(i)} + g_{k(ij)} + b_l + e_{m(ijkl)} \,, \qquad (4)$$

where s_i represents the ith sire, $d_{j(i)}$ the jth dam within sire. $g_{k(ij)}$ represents genotype in the breeding design (i.e., the 312 genotypes grown in the glasshouse) because there are multiple cuttings per genotype. b_l and $e_{m(ijkl)}$ represent experimental block and the residual, respectively. We used equation 4 to estimate **G** at each transplant elevation for the 2018 experiment. We then used the same analytical approach that we used for *S. aethnensis* (above) to test how genetic variance changed across elevation: First by comparing total genetic variance (the trace of **G**), and then by testing how much genetic variation the eigenvectors of **G** (at the 500m native site) describe at each elevation. We predicted that if we found the same patterns as presented in the main text of the current (2019) study, then it is unlikely that selection would have significantly affected estimates of genetic variance.

We found that genetic variance changed across elevation in the 2018 experiment in the same way to the current (2019) study (**Fig. S13**). Total genetic variance was lower at higher elevations (**Fig. S13a**), while axes of genetic variation were conserved across elevations (**Fig. S13b**). For *S. chrysanthemifolius,* genetic variance across elevation therefore involves greater changes in the amount of genetic variance compared to changes in orientation.

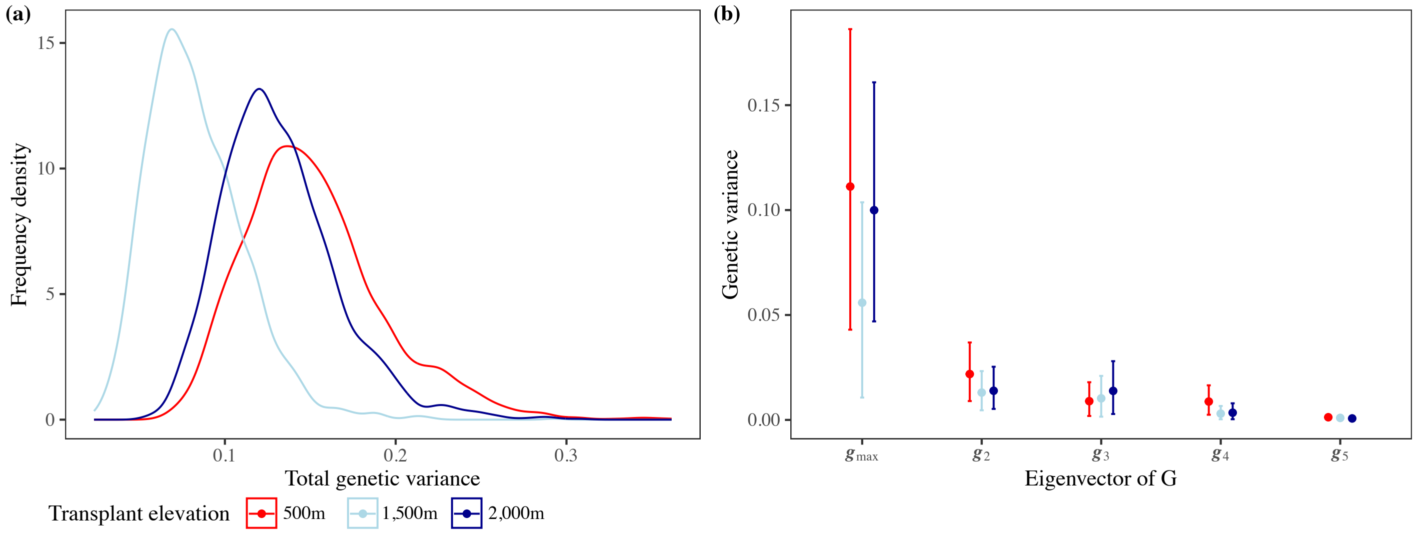

Fig. S13 Comparing genetic variance estimated before and after selection. **(a)** Estimates of total additive genetic variance are the same when estimated on individuals before selection (orange) versus surviving individuals after selection (blue). Density distributions represent the posterior distribution of the sum of the diagonal components of **G** (i.e., the trace) for each MCMC sample. **(b)** Eigenvectors of **G** (estimated before selection) describe the same amount of genetic variance in **G** estimated before and after selection. Credible intervals represent the 95% posterior distribution for the projection of each eigenvector for each MCMC sample of **G**.

Supplementary references

Lynch, M., and B. Walsh. 1998, Genetics and analysis of quantitative traits. Sunderland, Sinauer Associates, Inc.

Martin, G., E. Chapuis, and J. Goudet. 2008. Multivariate Q_{st}–F_{st} Comparisons: A Neutrality Test for the Evolution of the G Matrix in Structured Populations. Genetics 180:2135-2149.

Milner, J. M., J. M. Pemberton, S. Brotherstone, and S. D. Albon. 2000. Estimating variance components and heritabilities in the wild: a case study using the 'animal model' approach. Journal of Evolutionary Biology 13:804-813.

Schielzeth, H., and S. Nakagawa. 2013. Nested by design: model fitting and interpretation in a mixed model era. Methods in Ecology and Evolution 4:14-24.

Walter, G. M., J. Clark, D. Terranova, S. Cozzolino, A. Cristaudo, S. J. Hiscock, and J. R. Bridle. 2023. Hidden genetic variation in plasticity increases the potential to adapt to novel environments. New Phytologist 239:374-387.
